# Transcriptional Analysis and Subcellular Protein Localization Reveal Specific Features of the Essential WalKR System in *Staphylococcus aureus*

**DOI:** 10.1371/journal.pone.0151449

**Published:** 2016-03-21

**Authors:** Olivier Poupel, Mati Moyat, Julie Groizeleau, Luísa C. S. Antunes, Simonetta Gribaldo, Tarek Msadek, Sarah Dubrac

**Affiliations:** 1 Institut Pasteur, Biology of Gram-Positive Pathogens, Department of Microbiology, Paris, France; 2 CNRS, ERL3526, Paris, France; 3 Institut Pasteur, Molecular Biology of Gene in Extremophiles, Department of Microbiology, Paris, France; University of Manchester, UNITED KINGDOM

## Abstract

The WalKR two-component system, controlling cell wall metabolism, is highly conserved among Bacilli and essential for cell viability. In *Staphylococcus aureus*, *walR* and *walK* are followed by three genes of unknown function: *walH*, *walI* and *walJ*. Sequence analysis and transcript mapping revealed a unique genetic structure for this locus in *S*. *aureus*: the last gene of the locus, *walJ*, is transcribed independently, whereas transcription of the tetra-cistronic *walRKHI* operon occurred from two independent promoters located upstream from *walR*. Protein topology analysis and protein-protein interactions in *E*. *coli* as well as subcellular localization in *S*. *aureus* allowed us to show that WalH and WalI are membrane-bound proteins, which associate with WalK to form a complex at the cell division septum. While these interactions suggest that WalH and WalI play a role in activity of the WalKR regulatory pathway, deletion of *walH* and/or *walI* did not have a major effect on genes whose expression is strongly dependent on WalKR or on associated phenotypes. No effect of WalH or WalI was seen on tightly controlled WalKR regulon genes such as *sle1* or *saouhsc_00773*, which encodes a CHAP-domain amidase. Of the genes encoding the two major *S*. *aureus* autolysins, AtlA and Sle1, only transcription of *atlA* was increased in the Δ*walH* or Δ*walI* mutants. Likewise, bacterial autolysis was not increased in the absence of WalH and/or WalI and biofilm formation was lowered rather than increased. Our results suggest that contrary to their major role as WalK inhibitors in *B*. *subtilis*, the WalH and WalI proteins have evolved a different function in *S*. *aureus*, where they are more accessory. A phylogenomic analysis shows a striking conservation of the 5 gene *wal* cluster along the evolutionary history of Bacilli, supporting the key importance of this signal transduction system, and indicating that the *walH* and *walI* genes were lost in the ancestor of Streptococcaceae, leading to their atypical 3 *wal* gene cluster, *walRKJ*.

## Introduction

*Staphylococcus aureus*, a highly versatile bacterium, is successful both as a commensal as well as a life-threatening pathogen. This dual lifestyle is facilitated by its sophisticated arsenal of virulence factors and the regulatory networks controlling their production and its general metabolism, allowing it to rapidly adapt to host infection stress. Several global regulation systems allowing coordinated expression of virulence factors have been described in *S*. *aureus*. Among these, the well-known AgrCA quorum sensing two-component system, active at high cell density, positively controls the expression of genes encoding secreted host cell and extracellular matrix degradation proteins and represses genes for surface proteins involved in adhesion and escape from the immune response [1]. This reciprocal regulation is thought to allow early bacterial colonization at low bacterial densities, and wide bacterial spread when higher cell densities induce the AgrCA system.

Another two-component system, WalKR, is essential for cell viability and also plays a role in *S*. *aureus* virulence. This system is mainly involved in regulation of cell wall metabolism [2–4], a cellular process crucial for bacterial fitness that can lead to NFκB-dependent induction of the innate immune inflammatory response through release of cell wall degradation products that bind to the Nod2 receptor in the host [5, 6]. Increased activity of the WalKR system also indirectly induces genes involved in degradation of the cellular and extracellular host matrix through the SaeRS two-component system [6].

Genes encoding the WalKR system are located within the *wal* genetic locus, encompassing five genes in *S*. *aureus*: *walR*, *walK*, *walH*, *walI* and *walJ*. The WalKR system is highly conserved among Firmicutes, and its characterization in several bacterial species (*S*. *aureus*, *Bacillus subtilis*, *Streptococcus pneumoniae*) has highlighted its crucial role in controlling cell wall metabolism [3, 4, 7, 8]. This regulation has been shown to be responsible for the essential nature of the WalKR system in *S*. *pneumoniae* and *S*. *aureus* [9, 10]. The conservation of the *walR*, *walK*, *walH*, *walI* and *walJ* gene order among Bacilli suggests functional interactions between the corresponding proteins [11], although this has only been partially characterized. In *B*. *subtilis*, the WalH and WalI membrane-bound proteins were shown to interact with the WalK histidine kinase, inhibiting its activity [12, 13]. The *walJ* gene, encoding a β-lactamase fold protein, was first described as affecting colony morphology in *B*. *subtilis* [12]. Deletion of *walJ* has also been reported to cause a DNA segregation defect in *B*. *subtilis*, leading to the hypothesis that WalJ could be involved in coordinating cell division and DNA replication [14]. In *Bacillus anthracis*, interruption of *walJ* leads to a spontaneous mutator phenotype, and it has recently been described as a novel 5'-3' double-stranded DNA exonuclease playing a role in DNA mismatch repair [15–17]. In *Streptococcaceae*, the *wal* locus is reduced to only three genes, *walR*, *walK* and *walJ*, with the *walH* and *walI* genes absent from the genome. WalJ is required for optimal growth in *S*. *pneumoniae* when cells are partially depleted for WalKR, suggesting a functional link between WalJ and the WalKR system [9]. In *S*. *mutans*, WalJ appears to have a pleiotropic effect on growth, natural competence, oxidative stress tolerance and biofilm formation [18].

In *S*. *aureus*, the roles of WalH, WalI and WalJ had never been studied. We carried out a transcriptional analysis of the entire *wal* locus in order to characterize its genetic structure. Protein topology, protein-protein interactions and subcellular localization of the Wal proteins allowed us to show that WalH and WalI are membrane-bound proteins which associate with WalK to form a complex at the cell division septum. Phenotypic investigation of the Δ*walH* and Δ*walI* mutants indicate that WalH and WalI do not act as major negative regulators of WalKR activity as they do in *B*. *subtilis*. A detailed phylogenomic study indicates coevolution of the *wal* genes in Firmicutes with a concomitant loss of the *walH* and *walI* genes in *Streptococcaceae*.

## Materials and Methods

### Bacterial strains and media

*Escherichia coli* K12 strain DH5α™ (Invitrogen, Thermo Fisher Scientific, Waltham, MA) was used for cloning experiments. *Staphylococcus aureus* strain HG001 was used for genetic and functional studies. Plasmids were first transformed into the restriction deficient *S*. *aureus* strain RN4220 before introduction into the HG001 strain. Strains and plasmids are listed in Table 1. *E*. *coli* was grown in LB medium with ampicillin (100 μg/ml) and kanamycin (50 μg/ml) added when required. *S*. *aureus* was grown in Trypticase Soy Broth (TSB; Difco; Becton, Dickinson and Co., Franklin Lakes, NJ) supplemented with erythromycin (1 μg/ml) or chloramphenicol (10 μg/ml) when required. Expression from the P*cad* promoter was induced by adding cadmium chloride (CdCl_2_) at a final concentration of 0.25 μM.

**Table 1:** Bacterial strains and plasmids used in this study.

Strain or plasmid	Description	Source, reference or construction^a^
***E*. *coli* strains**		
DH5α	F^**-**^ Φ80*lac*ZΔM15 Δ(*lac*ZYA-*arg*F) U169 *rec*A1 *end*A1 *hsd*R17 (rK–, mK+) *pho*A *sup*E44 λ– *thi*-1 *gyr*A96 *rel*A1	Invitrogen Life Technology
DHT1	F^**-**^*glnV44*(AS) *recA1 endA1 gyrA96* (Nal^r^) *thi-1 hsdR17 spoT1 rfbD1 cya-854 ilv-691*::Tn*10*	[30]
***S*. *aureus* strains**		
RN4220	Restriction-deficient transformation recipient strain	[70]
HG001	NCTC 8325 *rsbU+*	[71]
ST1397	HG001Δ*walH*	pMAD*walH* → HG001
ST1130	HG001Δ*walI*	pMAD*walI* → HG001
ST1410	HG001Δ*walHI*	pMAD*walHI* → HG001
ST1131	HG001Δ*walJ*	pMAD*walJ* → HG001
ST1365	RN4220 P*walR*(RI+RII)-*lacZ*	pSA14-P*walR*(RI+RII) → RN4220
ST1366	RN4220 P*walR*(RI)-*lacZ*	pSA14-P*walR*(RII) → RN4220
ST1367	RN4220 P*walJ*-*lacZ*	pSA14-P*walJ* → RN4220
ST1398	RN4220 pSA14	pSA14→ RN4220
ST1301	HG001 *walH*-GFP	pOLSA-*walH* → HG001
ST1340	HG001 *walK*-GFP	pOLSA-*walK* → HG001
ST1341	HG001 *walI*-GFP	pOLSA-*walI* → HG001
ST1378	HG001 *walJ*-GFP	pOLSA-*walJ* → HG001
ST1415	HG001Δ*walH* pMK4Pprot-*walHI*	pMK4Pprot-*walHI* → ST1397
ST1416	HG001Δ*walI* pMK4Pprot-*walHI*	pMK4Pprot-*walHI* → ST1130
ST1417	HG001Δ*walHI* pMK4Pprot-*walHI*	pMK4Pprot-*walHI* → ST1410
**Plasmids**		
pMAD	Allelic exchange vector	[19]
pMAD*walH*	pMAD derivative for *walH* deletion	This study
pMAD*walI*	pMAD derivative for *walI* deletion	This study
pMAD*walHI*	pMAD derivative for simultaneous *walH* and *walI* deletion	This study
pMAD*walJ*	pMAD derivative for *walJ* deletion	This study
pMAD*walK*	pMAD derivative for *walK* deletion	This study
pSA14	Vector for constructing transcriptional *lacZ* fusions	[22]
pSA14-P*walR*(RI+RII)	pSA14 derivative carrying the full-length *walRKHI* promoter region (promoters PI and PII)	This study
pSA14-P*walR*(RII)	pSA14 derivative carrying the *walRKHI* PII promoter region	This study
pSA14-P*walJ*	pSA14 derivative carrying the *walJ* promoter region	This study
pCN51	Vector for CdCl_2_-dependent gene expression	[31]
pOLSA	Vector for expression of GFP translational fusions	This study
pOLSA-*walK*	pOLSA derivative containing the entire *walK* coding sequence in frame with GFP	This study
pOLSA-*walH*	pOLSA derivative containing the entire *walH* coding sequence in frame with GFP	This study
pOLSA*walI*	pOLSA derivative containing the entire *walI* coding sequence in frame with GFP	This study
pOLSA*walJ*	pOLSA derivative containing the entire *walJ* coding sequence in frame with GFP	This study
pKTop	Vector expressing the dual reporter PhoA21-471/LacZ5-60, p15 ori	[26]
pKTop-*walH*	pKTop derivative for expression of WalH1-40/PhoA/LacZ protein fusion	This study
pKTop-*walI*	pKTop derivative for expression of WalI1-40/PhoA/LacZ protein fusion	This study
pKTop-*walJ*	pKTop derivative for expression of WalJ1-40/PhoA/LacZ protein fusion	This study
pKT25	BACTH vector designed to express a given polypeptide fused in frame at its N-terminal end with the CyaA T25 fragment; p15 ori	[29]
pKT25-Zip	Zip-Zip BACTH positive control vector	[29]
pKT25-*walR*	Full-length *walR* cloned into pKT25	This study
pKT25-*walK*	Full-length *walK* cloned into pKT25	This study
pKT25-*walH*	Full-length *walH* cloned into pKT25	This study
pKT25-*walI*	Full-length *walI* cloned into pKT25	This study
pKT25-*walJ*	Full-length *walJ* cloned into pKT25	This study
pUT18c	BACTH vector designed to express a given polypeptide fused in frame at its N-terminal end with the CyaA T18 fragment; ColE1 ori	[29]
pUT18c-Zip	Zip-Zip BACTH positive control vector	[29]
pUT18c-*walR*	Full-length *walR* cloned into pUT18c	This study
pUT18c-*walK*	Full-length *walK* cloned into pUT18c	This study
pUT18c-*walH*	Full-length *walH* cloned into pUT18c	This study
pUT18c-*walI*	Full-length *walI* cloned into pUT18c	This study
pUT18c-*walJ*	Full-length *walJ* cloned into pUT18c	This study
pMK4-Pprot	pMK4 derivative carrying a constitutive Gram-positive promoter for gene complementation	[21]
pMK4-Pprot*walHI*	pMK4-Pprot derivative carrying *walHI*	This study

^a^ Arrows indicate plasmid introduction by electroporation

### Mutant construction and complementation

Oligonucleotides used in this study were synthesized by Eurofins Genomics (Ebersberg, Germany) and their sequences are listed in Table 2. Gene deletions were performed using the thermosensitive replication vector pMAD carrying DNA fragments of approximately 800 bp corresponding to the upstream and downstream regions flanking the gene of interest [19]. The cloned DNA fragments were amplified by PCR using chromosomal DNA from strain HG001 and the oligonucleotide pairs listed in Table 2. Given the close proximity/overlap of the *walK*, *walH* and *walI* genes and their likely translational coupling, oligonucleotides for gene deletions were carefully designed so as to preserve translation initiation signals and avoid any polar effects on the distal genes. Oligonucleotide pairs used for amplifying DNA fragments for gene deletions were as follows: Δ*walH* (OP238/OSA528 and OSA529/OP241); Δ*walI* (OP242/OP243 and OP244/OP245); Δ*walHI* (OP238/OSA528 and OP244/OP245); Δ*walJ* (OP201/OP202 and OP203/OP204); Δ*walK* (in the Δ*walHI* mutant, OP369/OP370 and OP244/OP245). The resulting plasmids were then introduced into *S*. *aureus* at a permissive temperature (30°C) and integration/excision cycles were induced by temperature shifts (30°C-37°C) as previously described [19]. Candidate mutants were then tested by PCR.

**Table 2:** Oligonucleotides used in this study.

Name	Sequence	Description
***Construction of pMAD derivatives***		
OP238	TGCAGGATCCAGGATTTGTAACTGGTTATA	*walH* upstream fragment (*Bam*HI/*Eco*RI)
OSA528	ATGGAATTCATTATTCATCCCAATCACCGTCTTC	
OSA529	TATGAATTCGGGAGGCTTGAATAAATGAACTGG	*walH* downstream fragment (*Eco*RI/*Nco*I)
OP241	ATTCTCCATGGGATTCATATTAATGATTAATAATTTTAG	
OP242	TGCAGGATCCATGCAACCATACACAGATATCATCACA	*walI* upstream fragment (*Bam*HI/*Eco*RI)
OP243	AGCCGAATTCTTATTCAAGCCTCCCATCGTTATAAAC	
OP244	AGCCGAATTCTATGAATCGTAATAAGCTAGTATTGCA	*walI* downstream fragment (*Eco*RI/*Nco*I)
OP245	ATTCTCCATGGTGCGGATCTATTGCATCATGTGAC	
OP201	ATTCTGGATCCGTAAAATACGAACAAACGT	*walJ* upstream fragment (*Bam*HI/*Eco*RI)
OP202	AGCCGAATTCCCTTTCTCTTTAAACAGTCA	
OP203	ATGCATGAATTCAAATGAGAGTCACCCTAT	*walJ* downstream fragment (*Eco*RI/*Bgl*II)
OP204	GTGGTTAGATCTTGGAAGGCGTCTCCTGCG	
OP369:	TGCAGGATCCCCGATTGCTGATATTTTAGAAT	*walK* upstream fragment
OP370	AGCCGAATTCTCGTTTCGACCTCTACTCATGT	(*Bam*HI/*Eco*RI)
***Complementation of wal mutants***		
OP277	TGCAGGATCCTGAAGTCATTGAAGACGGTGATT	*walHI* coding sequences
OSA542	CTCTGCAGTGATGAGCTTGCAATGCTAGCTTATTACG	(*Bam*H1/*Pst*I)
***Construction of transcriptional lacZ fusions***		
OSA477	AATCTGCAGTAGTAATATATAAGTTTATATTGG	*walR* upstream promoter region, PI + PII, forward (*Pst*I)
OSA478	AATCTGCAGAATAGTAAGCGACATCCTGTG	*walR* upstream promoter region, PII, forward (*Pst*I)
OSA473	TCTGGATCCTTGCATAAACCTCTTTTCTTAAATC	*walR* upstream region, reverse (*Bam*HI)
OSA479	TTACTGCAGATGTTTTTGCGTCTCCAACGTG	*walJ* upstream region, forward (*Pst*I)
OSA475	GCGGGATCCCCTTTCTCTTTAAACAGTCAATC	*walJ* upstream region, reverse (*Bam*HI)
***Construction of pKTop derivatives***		
OMA16	CGGATCCATGTGAAGTCATTGAAGACGGTGATTGGG	WalH (AA 1 to 40*)* coding sequence (*Bam*HI/*Kpn*I)
OMA17	TCGGTACCCTATCTGTATTGTCGACATTTGCAATATCAGG	
OMA12	TGGATCCTTTATAACGATGGGAGGCTTGAATAAATG	WalI (AA 1 to 40*)* coding sequence (*Bam*HI/*Kpn*I)
OMA13	ACGGTACCTTGTTACTCTCGACTTCATTAATGTGTGAGCG	
OMA14	CGGATCCATAGAGAGATTGACTGTTTAAAGAGAAAGG	WalJ (AA 1 to 40*)* coding sequence (*Bam*HI/*Kpn*I)
OMA15	AAGGTACCTCTTCCATTTTCTTTCCAGTCAAACCAACATC	
***Construction of pOLSA and derivatives***		
OSA338	CCCCCGGGTCAGGGTCAGGGTCAAAAGGAGAAGAATTATTTACAGGGG	Peptide linker and GFPopt coding sequence (*Xma*I/*Kpn*I)
OSA339	AAGGTACCTTACTTATATAATTCATCCATTCCG	
OSA340	AAGGATCCGAGTAGAGGTCGAAACGAATGAAGTGGC	WalK coding sequence (*Bam*HI/*Xma*I)
OSA341	CCCCCGGGTTCATCCCAATCACCGTCTTCAATGACTTCAC	
OSA357	TGGGATCCTTGAAGACGGTGATTGGGATGAATAATAAGG	WalH coding sequence (*Bam*HI/*Xma*I)
OSA358	TTCCCGGGTTCAAGCCTCCCATCGTTATAAACATACC	
OMA12	TGGATCCTTTATAACGATGGGAGGCTTGAATAAATG	WalI coding sequence (*Bam*HI/*Xma*I)
OMA34	TCCCCGGGATGATTAATAATTTTAGGGTTATTATTTGTCG	
OMA14	CGGATCCATAGAGAGATTGACTGTTTAAAGAGAAAGG	WalJ coding sequence (*Bam*HI/*Xma*I)
OMA35	CTCCCGGGTATTGTATATATTGGCGTTGGAATAGC	
***Construction of BACTH plasmids***		
OSA361	TTGGATCCCATGGCTAGAAAAGTTGTTGTAGTTGATGATG	WalR coding sequence (*Bam*HI/*Kpn*I)
OSA362	TTGGTACCTTTCGACCTCTACTCATGTTGTTGG	
OSA363	TCGGATCCCATGAAGTGGCTAAAACAACTACAATCCC	WalK coding sequence (*Bam*HI/*Kpn*I)
OSA364	AGGGTACCTATGCTCCTTATTATTCATCCC	
OSA365	GTGGATCCCATGAATAATAAGGAGCATATTAAATCTG	WalH coding sequence (*Bam*HI/*Kpn*I)
OSA366	TGGGTACCTCAGTTTCCAGTTCATTTATTCAAGCC	
OSA367	CTGGATCCCATGAACTGGAAACTGACAAAGACAC	WalI coding sequence (*Bam*HI/*Kpn*I)
OSA368	TTGGTACCATTCATATTAATGATTAATAATTTTAGGG	
OSA369	AGGGATCCCATGAGCCGCTTGATACGCATGAGTG	WalJ coding sequence (*Bam*HI/*Kpn*I)
OSA370	TTGGTACCATGACTCTCATTTATATTGTATATATTGGC	
***Reverse transcription PCR analysis***		
OJU20	TATTGTGACGCGTAGAGGCGTTGG	3' part of *walR*, forward
OJU21	CTTGACGGTTGGCATACTCACT	5' part of *walK*, reverse
OJU22	CGCACAATGGTCGTATTTGGGC	3' part of *walK*, forward
OJU23	CGAATGAATAATCTGAAATGGC	5' part of *walH*, reverse
OJU24	GAAGTGCAGATTAACAGTGAACTCG	3' part of *walH*, forward
OJU25	TCGACTTCATTAATGTGTGAGCG	5' part of *walI*, reverse
OJU1	CGGTTACTACTCAGTCGTGAATGAAACG	3' part of *walI*, forward
OP227	CTTGAATATTACGGTCAATTTGACT	5' part of *walJ*, reverse
OJU17	TTCATTGCACGTGATTATC	Intergenic *walI-walJ* region, forward
***Primer extension experiments***		
OSA433	CTTATAAATGGTAAATTATATAATAGG	*walR* upstream region, forward
OSA434	GTTAAATTCTAAAATATCAGCAATCGG	*walR* coding sequence, reverse
OSA435	TTTGCTGGTACGGTTTATTATGGC	*walJ* upstream region, forward
OSA437	TAATACACTCATGCGTATCAAGCG	*walJ* coding sequence, reverse
***qRT-PCR experiments***		
OSA161	ACGTGGATAACCTACCTATAAGACTGGGAT	*16S rRNA*
OSA162	TACCTTACCAACTAGCTAATGCAGCG	
OSA138	GTGTACTGTGCATACGATGGTAATGATGC	*walR*
OSA139	CGTTACATAGTCATCTGCACCTAGTTCTA	
OSA203	AACAGCACCAACGGATTAC	*atlA*
OSA204	CATAGTCAGCATAGTTATTCATTG	
OSA209	AATTATATTCATACAATCCTGGTG	*saouhsc-00773*
OSA210	GGTGCTTGCTTAACTACTAC	
OSA224	AAGTATCTGGCTCAAGTAATTCTAC	*sle1*
OSA225	TGATGGACGGCTACTATTGC	

Plasmid pMK4Pprot, a derivative of shuttle vector pMK4 [20] carrying a constitutively expressed Gram-positive promoter sequence [21] was used for gene complementation experiments. Complementation of the ST1397 (Δ*walH*), ST1130 (Δ*walI*) and ST1410 (Δ*walHI*) strains was carried out using a 2204 bp DNA fragment corresponding to the complete *walHI* coding sequences, amplified with oligonucleotides OP277/OSA542, generating *Bam*HI/*Pst*I restriction sites at the extremities, and cloned in the replicative plasmid pMK4-Pprot, to give plasmid pMK4Pprot-*walHI*.

Nucleotide sequencing of plasmid constructs was carried out by Beckman Coulter Genomics (Danvers, MA).

### β-Galactosidase assays

Plasmid pSA14 [22] is a derivative of shuttle vector pMK4 [20], carrying a promoterless *E*. *coli lacZ* gene and was used to construct transcriptional *lacZ* reporter fusions. Promoter regions of the *walRKHI* operon and the *walJ* gene were amplified by PCR using oligonucleotides introducing *Pst*I/*Bam*HI restriction sites (see Table 2). The DNA fragments were cloned between the corresponding restriction sites of the pSA14 vector, yielding plasmids pSA14-P*walR*(RI+RII), pSA14-P*walR*(RII) and pSA14-P*walJ* (Table 1).

For β-galactosidase assays in *S*. *aureus*, cells carrying *lacZ* fusions were grown in TSB until OD_600 nm_ ≈ 3 and harvested by centrifuging 2 ml culture samples (2 min; 5,400 x *g*). Assays were performed as previously described [23] and β-galactosidase specific activities expressed as Miller units mg^−1^ protein [24]. Protein concentrations were determined using the Bio-Rad protein assay (BioRad, Hercules, CA) [25].

### Protein topology analysis

To study the topology of the WalH, WalI and WalJ proteins, we used the pKTop plasmid carrying the dual *pho-lac* reporter gene, which encodes the *E*. *coli* alkaline phosphatase fragment PhoA_21-471_, fused in-frame with the α-peptide of *E*. *coli* β-galactosidase, LacZ_4-60_, allowing protein topology determination *in vivo* [26]. Extracellular or membrane localization of the reporter leads to high alkaline phosphatase activity and low β-galactosidase activity, whereas a cytosolic localization results in high β-galactosidase activity and low alkaline phosphatase activity. A strain carrying the pKTOP vector alone, in the absence of any inserts, will exhibit β-galactosidase activity, but no alkaline phosphatase activity, as there are no secretion signals for the ‘PhoA’-‘LacZ’ fusion [26]. Transmembrane domain predictions were carried out using the Phobius web server (http://phobius.sbc.su.se; [27]). Sequences encoding the first 40 amino acids, encompassing the predicted transmembrane domain, were amplified by PCR using oligonucleotides OMA16/OMA17 for *walH* and OMA12/OMA13 for *walI* (Table 2). The WalJ protein, predicted to be cytosolic, was also included in this study, and a DNA fragment corresponding to the first 40 codons was amplified using oligonucleotides OMA14/OMA15 (Table 2). These DNA fragments were cloned between the *BamH*I and *Kpn*I restriction sites of pKTop, resulting in translational fusions with the dual *pho-lac* reporter genes.

Protein topology assays were carried out using *E*. *coli* strain DH5α on dual-indicator LB agar plates containing isopropyl-β-D-thiogalactopyranoside (IPTG; 1 mM); 6-chloro-3-indolyl-β-D-galactopyranoside (Red-Gal; Sigma-Aldrich, St. Louis, MO) at 100 μg/ml (for β-galactosidase activity) and 5-bromo-4-chloro-3-indolyl phosphate disodium salt (X-Pho; Sigma-Aldrich) at 80 μg/ml (for alkaline phosphatase activity), as previously described [28].

### BACTH protein interaction assays

For testing protein interactions using the Bacterial Adenylate Cyclase Two-Hybrid System (BACTH), genes encoding the proteins of interest were cloned into plasmids pKT25 and pUT18c leading to translational fusions with the T25 and the T18 domains of the *Bordetella pertussis* adenylate cyclase [29]. DNA fragments were amplified by PCR using chromosomal DNA from strain HG001 and specific oligonucleotides (see Table 2) and digested with *BamH*I and *Kpn*I for introduction into pKT25 and pUT18c. The resulting plasmids were then co-transformed into *E*. *coli* strain DHT1 [30] to detect protein interactions and transformants were selected on kanamycin (50 μg/ml) for pKT25 derivatives and ampicillin (100 μg/ml) for pUT18c derivatives.

*E*. *coli* strain DHT1 transformants carrying combinations of pKT25 and pUT18c derivatives were tested for cyclic AMP-dependent activation of *lacZ* expression. Strains were grown in LB medium supplemented with ampicillin (100 μg/ml) and kanamycin (50 μg/ml). Overnight cultures were then spotted on LB-agar plates containing isopropyl β-D-1-thiogalactopyranoside (IPTG; 0.5 mM), ampicillin (100 μg/ml), kanamycin (50 μg/ml), and 5-bromo-4-chloro-3-indolyl-β-D-galactopyranoside (X-Gal; 100 μg/ml). Plates were incubated for 24 H at 30°C and examined for the characteristic blue color indicative of β-galactosidase activity through X-Gal hydrolysis. Quantitative β-galactosidase assays were performed on exponentially growing *E*. *coli* liquid cultures (30°C, OD_600 nm_ ≈ 1), on SDS/chloroform permeabilized cells as previously described [24]. Zip-Zip associations were used as positive controls [29].

### Subcellular localization of fluorescent fusion proteins

The pOLSA plasmid was used to produce fluorescent GFP fusion proteins and is derived from the pCN51 vector carrying the P*cad-cadC* promoter module (cadmium chloride-inducible promoter and CadC repressor gene) [31]. A DNA fragment encoding an optimized GFP (GFPopt) preceded by a six amino acid peptide linker (PGSGSG) was cloned downstream from the P*cad* promoter in order to obtain a vector allowing production of a fluorescent fusion protein under cadmium-dependent expression. The GFPopt gene was amplified by PCR from the pTetONGFPopt plasmid [32] using oligonucleotides OSA338 and OSA339. Oligonucleotide OSA338 has a 5' extension encoding a PGSGSG peptide linker and allowing in frame translation with the downstream GFPopt. The amplified fragment was cloned into the pCN51 vector between the *Xma*I and the *Kpn*I restriction sites. Translational fusions of the WalK, WalH, WalI and WalJ proteins with GFPopt were constructed by PCR amplification using HG001 chromosomal DNA and the OSA340/OSA341, OSA357/OSA358, OMA12/OMA34, and OMA14/OMA35 oligonucleotide pairs, respectively (Table 2). Amplicons were cloned into pOLSA between the *BamH*I and *Xma*I restriction sites, allowing transcription from the P*cad* promoter and production of fusion proteins composed of the protein of interest, the peptide linker, and GFPopt.

Subcellular protein localizations were performed in HG001 *S*. *aureus* strains transformed with pOLSA derivatives producing GFP fusion proteins of interest. Fluorescence microscopy was carried out on cells grown in liquid cultures in TSB supplemented with CdCl_2_ (0.25 μM) to induce gene fusion expression. When cells reached OD_600nm_ ≈ 1.5 (exponential growth phase), they were harvested and concentrated 20 times in PBS. Cell suspensions were mixed with Vectashield mounting media (Vector Laboratories, Burlingame, CA) and used for microscopic observations with a Nikon Eclipse E600. Images were acquired with a Nikon Digital Camera DXM1200F. ImageJ software was used for quantifying fluorescence (http://imagej.nih.gov/ij/index.html; [33]). Fluorescence ratios were calculated by measuring fluorescence at the division septa divided by the fluorescence at the lateral wall after subtracting background fluorescence. Quantification was performed for 22 cells (11 septa) for each strain and the results were statistically analyzed by the Wilcoxon signed-rank test using GraphPad Prism 5.0d software (GraphPad Software, San Diego, CA; http://www.graphpad.com) with a *P* value < 0.05 considered significant.

### Bacterial autolysis assays

Bacteria were grown in TSB at 37°C with shaking until OD_600 nm_ ≈ 1, pelleted (10 min; 5,400 x *g*), resuspended in phosphate buffered saline (PBS) with Triton X-100 (0.1%), and incubated at 37°C with shaking. Lysis was determined as the decrease in OD_600 nm_ over time and indicated as a percentage of the initial OD (measured OD_600 nm_ / initial OD_600 nm_).

### Biofilm formation assays

Strains were grown overnight in TSB, diluted (1/1000) in TSB with glucose (0.75%), NaCl (3.5%) and distributed in PVC microtiter plates (200 μl per well). After 24 h incubation at 37°C, adherent biomass was rinsed twice with PBS, stained with crystal violet, resuspended in ethanol-acetone (80:20) and quantified by measuring OD_595 nm_, normalized to the OD_600 nm_ of each culture.

### Total RNA extraction

Bacteria were grown in TSB at 37°C with shaking and harvested once they reached OD_600 nm_ = 1 (2 min, 5,400 x *g*). RNA extractions were then performed as previously described [34], followed by a DNase I treatment with the TURBO DNA-free reagent (Ambion, Austin, TX) to eliminate residual genomic DNA.

### cDNA synthesis and quantitative real time PCR (qRT-PCR)

Complementary DNAs (cDNAs) were synthesized using the iScript cDNA synthesis kit (Bio-Rad, Hercules, CA), in a 20 μl reaction volume containing 1 μg total RNA. Oligonucleotides were designed for 100–200 bp amplicons using BEACON Designer 4.02 software (Premier Biosoft International, Palo Alto, CA) (see Table 2). qRT-PCRs, critical threshold cycles (CT) and *n*-fold changes in transcript levels were performed and determined as previously described [3] using the SsoFast^TM^ EvaGreen Supermix (Bio-Rad, Hercules, CA) and normalized with respect to 16S rRNA whose levels did not vary under our experimental conditions. All assays were performed using quadruplicate technical replicates, and repeated with three independent biological samples, and the data are presented as the mean and standard deviation.

### Reverse transcription PCR (RT-PCR)

We used RT-PCR to characterize the *wal* locus transcripts. Specific *wal* transcript cDNAs were synthesized from DNase I treated RNA samples (20 μg of total RNA) using reverse oligonucleotides OJU21 (*walK*), OJU23 (*walH*), OJU25 (*walI*), and OP227 (*walJ*) and AMV Reverse Transcriptase (Roche, Basel, Switzerland) according to the manufacturer’s recommendations. Specific cDNAs were then used as templates to test for co-transcription of *wal* genes by PCR amplification using *Taq* DNA polymerase (MP Biomedicals, Santa Ana, CA) with the following oligonucleotide pairs: OJU20/OJU21 to amplify the *walR-walK* junction, OJU22/OJU23 for the *walK-walH* junction, OJU24/OJU25 for the *walH-walI* junction; OJU1/OP227 for the *walI-walJ* junction, and OJU17/OP227 as a control to amplify the *walJ* 5' region (see Table 2 for oligonucleotide sequences). Absence of genomic DNA in RNA preparations was verified in control reactions omitting the reverse transcription step. Positive controls were obtained by using HG001 genomic DNA as the template. RT-PCR products were visualized after electrophoresis in 1.5% agarose gels with ethidium bromide staining.

### Primer extension analysis

Oligonucleotide primers (OSA434 for *walR*, and OSA437 for *walJ*) were 5’end-labeled with [γ-^32^P] ATP (Perkin Elmer, Waltham, MA) using T4 polynucleotide kinase (New England Biolabs, Ipswich, MA) and used in primer extension reactions with AMV reverse transcriptase (Roche). Briefly, 20 μg of total RNA and 1 pmol of labeled oligonucleotide were hybridized by heating to 65°C for 3 min and cooling to room temperature. The hybridized oligonucleotide was then extended with 10 U of AMV reverse transcriptase for 30 min at 42°C in the presence of all four dNTPs. The reaction was stopped by addition of formamide stop solution and subjected to electrophoresis on DNA sequencing gels alongside the corresponding dideoxy chain termination sequencing reactions with the same oligonucleotide. DNA sequencing was performed with the Sequenase PCR Product Sequencing Kit (USB, Cleveland, OH) using DNA fragments corresponding to the upstream regions of the genes, generated by PCR using oligonucleotide pairs OSA433/OSA434 for *walR* and OSA435/OSA437 for *walJ*.

### Phylogenomic analysis

A reference species tree was built from a concatenation of 47 widely distributed ribosomal proteins from 119 representatives of the Bacilli class plus 24 representatives of the Clostridia class as an outgroup. Exhaustive homology searches of the complete set of ribosomal proteins from Firmicutes were performed by BlastP, TBlastN, and different seeds on a local database of complete Firmicute genomes obtained from the NCBI. Each protein data set was aligned using Muscle [35] with default parameters, and unambiguously aligned positions were automatically selected using the BMGE software for multiple-alignment trimming [36] with a BLOSUM30 substitution matrix. Trimmed alignments were then concatenated by allowing a maximum of 17 missing proteins per data set, giving a final character supermatrix of 47 ribosomal proteins and 5,945 amino acid positions for phylogenetic analysis. A maximum likelihood tree was calculated by RAxML [37], with the PROTCATLGF model and four categories of evolutionary rates, as suggested by the Akaike information criterion (AIC) implemented in Treefinder [38]. Support at nodes was calculated by nonparametric bootstrap from 1000 resamplings of the original alignment.

A search for homologs of the WalR-K-H-I-J proteins followed the same strategy as described above. Single gene trees were obtained by RAxML and showed nearly identical topologies, justifying concatenation. Assembly of a concatenated data set followed the same strategy detailed above, by allowing a maximum of 17 missing proteins per data set giving a final character supermatrix of 1,102 amino acid positions. A maximum likelihood tree was calculated by RAxML as described above. Wal protein accession numbers are listed in S1 Table.

## Results

### The *S*. *aureus wal* locus consists of two separate transcription units

The *wal* locus contains 5 genes in *S*. *aureus*, *walR* and *walK*, encoding the essential WalKR two-component system (TCS), followed by *walH*, *walI* and *walJ*. Sequence inspection of the *S*. *aureus wal* locus indicates that the *walK* ATG is only 12 bp downstream from the *walR* amber stop codon, which overlaps the *walK* ribosome-binding site. Likewise, the *walK* and *walH* coding sequence have an 8 bp overlap with the *walK* ochre codon located within the beginning of the *walH* coding sequence (ATGaaTAA), whereas the *walH* ochre codon is immediately followed by the *walI* ATG codon. We noted that the intergenic region between *walI* and *walJ* is particularly long in *S*. *aureus*, 389 bp, compared to only 21 bp in *B*. *subtilis*. These observations suggest that *walJ* may have evolved to be transcribed independently from the other *wal* genes in *S*. *aureus*, and that *walRKHI* are cotranscribed as an operon, with translational coupling between all four genes leading to stoichiometric production of WalR, WalK, WalH and WalI.

**Fig 1:**
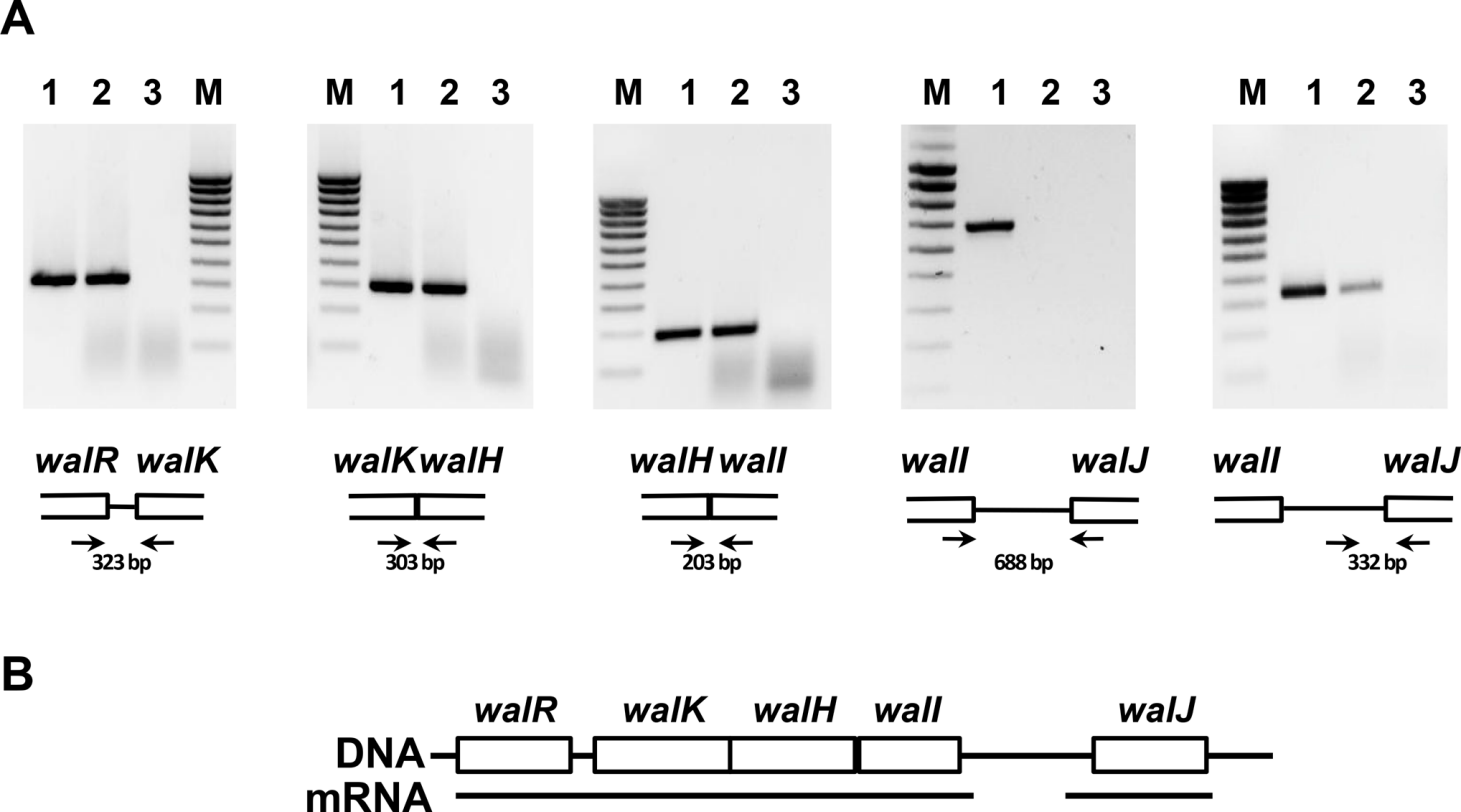
The *S*. *aureus wal* locus consists of two separate transcription units. (A) *S*. *aureus* total RNA was used to synthesize cDNAs and amplify intergenic regions with oligonucleotides hybridizing specifically within the upstream and downstream genes (indicated by arrows, not to scale). DNA fragments were separated by electrophoresis on ethidium bromide stained 1.5% agarose gels and their size is indicated. Lanes 1: positive PCR control using HG001 genomic DNA as the template; lanes 2: PCR using cDNA as the template; lanes 3: control PCR reaction using total RNA as the template (without reverse transcriptase treatment). M: 100 bp molecular mass ladder (Eurogentec, Angers, France). (B) Representation of the *wal* locus and the corresponding transcripts (not to scale).

This prompted us to carry out a transcriptional analysis of the *wal* locus in *S*. *aureus*. Total RNA was extracted from mid-exponential phase cultures (OD_600 nm_ = 1) in rich medium (TSB) and used for RT-PCR experiments with specific primers designed to amplify DNA fragments encompassing the junctions between each of the *wal* locus genes (see Table 2 and Fig 1A). As shown in Fig 1A, using *wal* transcript cDNAs as a template, we were able to amplify DNA fragments corresponding to the junctions of *walR*/*walK*, *walK*/*walH* and *walH*/*walI*, indicating that these four genes are co-transcribed as a tetra-cistronic operon (Fig 1B). However, there was no amplification from cDNA using primers designed to amplify the *walI/walJ* junction, in agreement with our sequence based prediction that *walJ* is transcribed as an independent transcription unit. Positive PCR controls using chromosomal DNA as the matrix led to a DNA fragment corresponding to the *walI/walJ* intergenic region, indicating that the primers hybridize efficiently. Using the *wal* transcript cDNAs as the matrix, we were able to amplify a DNA fragment using oligonucleotides hybridizing within the *walJ* 5’ UTR and the beginning of the *walJ* coding sequence (Fig 1A, last panel), confirming that *walJ* mRNA was not degraded in the RNA preparation.

Taken together, our results indicate that *walJ* is transcribed as a monocistronic transcription unit whereas *walRKHI* are transcribed as an operon (Fig 1B). Indeed, sequence analysis indicates that the *walI* ochre codon lies immediately upstream from a probable rho-independent transcription terminator inverted repeat sequence followed by a poly(T) stretch (AAGCTAGCAttGCaagctcatcatatgtgagaaGCggTGCTAGCTT; ΔG = -98 kJ mol^-1^). Likewise, a potential rho-independent transcription terminator followed by a poly(T) stretch is located downstream from the *walJ* ochre codon (CAtCCGATAAAGTtccgcattgctgtgagacgACTTTATCGGgTG; ΔG = -85.3 kJ mol^-1^).

### The *walRKHI* operon and the *walJ* gene are transcribed from σ^A^-dependent promoters

**Fig 2:**
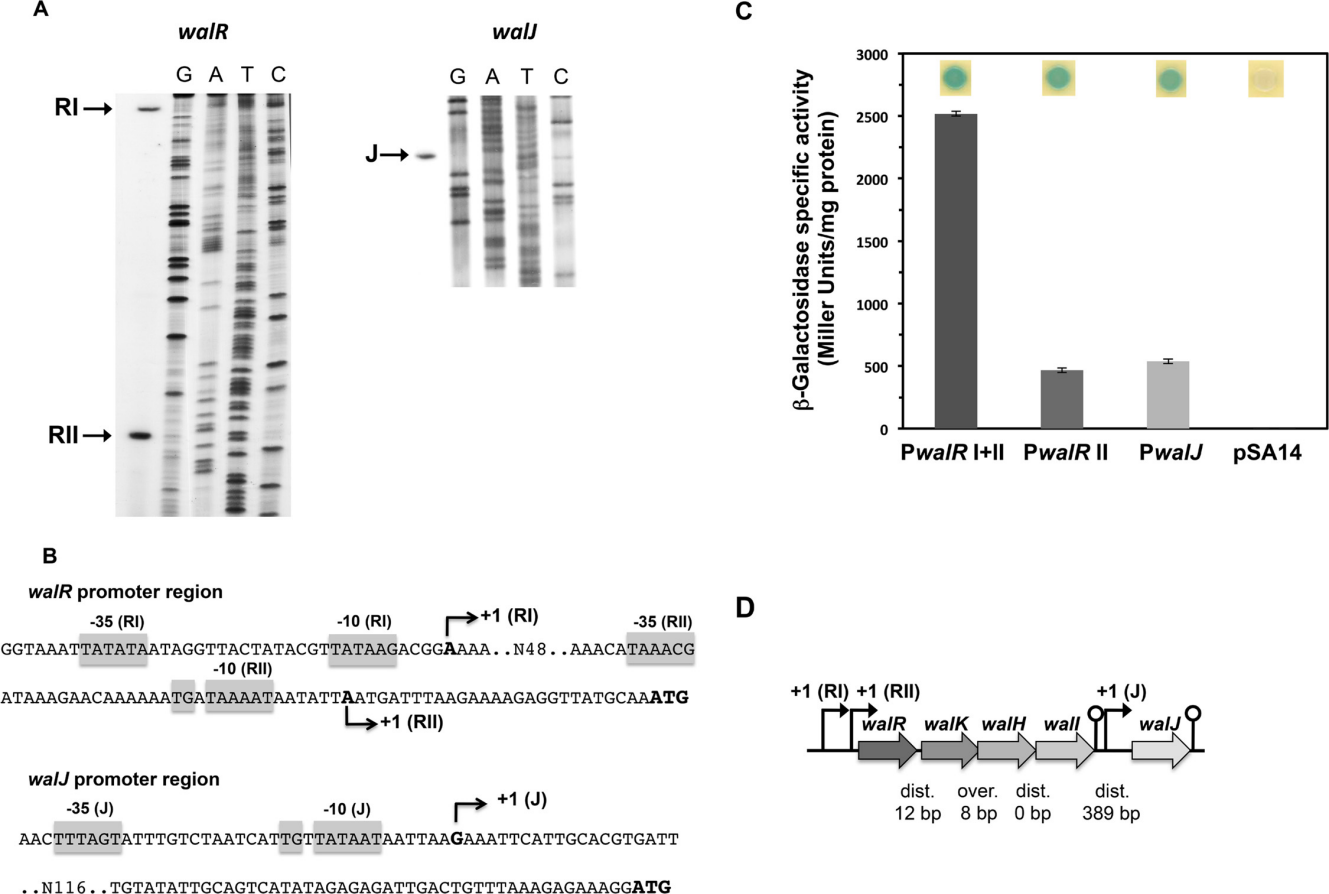
The *walRKHI* operon and the *walJ* gene are transcribed from σ^A^ promoters. (A) Primer extension analysis of the *walR* (left panel) and *walJ* (right panel) mRNA. Sanger dideoxy chain termination sequencing reactions (GATC) were carried out on PCR-generated DNA fragments corresponding to the respective upstream regions. (B) DNA sequence of the *walR* and *walJ* upstream regions with the identified transcription start sites indicated by arrows and consensus -10 and -35 sequences and extended -10 promoter TG dinucleotides shown by grey boxes. Translation and transcription initiation sites are indicated in bold letters. (C) Expression of the *walRKHI* operon and the *walJ* gene was followed using *lacZ* transcriptional fusions in *S*. *aureus* strains ST1365 (P*walR* RI + RII), ST1366 (P*walR* RII) and ST1367 (P*walJ*) and control strain ST1398 carrying plasmid pSA14 alone. β-Galactosidase assays were performed as described in Materials and Methods and measured during late exponential growth at 37°C in TSB. Cultures were spotted on TSB plates containing X-Gal as shown above the histogram bars. (D) Schematic representation of the *wal* locus genetic structure. Transcription start sites, nucleotide distances or overlap between coding sequences and transcription terminators downstream from *walI* and *walJ* are shown.

To further characterize transcription of the *wal* locus, we determined the transcriptional start sites for the *walRKHI* operon and the *walJ* gene. Primer extension experiments were performed with specific oligonucleotides hybridizing within the *walR* or *walJ* coding sequences. As shown in Fig 2A, with the *walR* oligonucleotide, two cDNAs were synthesized by primer extension (RI and RII). The 3’ end of the longer cDNA (RI) corresponds to a distal transcription start site located 119 bp upstream from the *walR* translational start site. It is preceded by appropriately spaced potential -10 (TATAAG) and -35 (TATATA) regions, sharing similarities with the consensus sequences of promoters recognized by the vegetative form of RNA polymerase holoenzyme, Eσ^A^ (Fig 2B). The shorter cDNA, whose 3’ end is 28 bp upstream from the *walR* translation initiation codon, could correspond either to mRNA processing or to transcription initiation from a secondary promoter. In front of this potential proximal transcriptional start site, there is a well-conserved -10 box (TAAAAT) preceded by a possible -35 sequence (TAAACG), and we noted that the -10 hexamer is directly preceded by a TG dinucleotide (Fig 2B) suggesting that this promoter belongs to the extended -10 promoter family, which are active even in the absence of a consensus -35 sequence [39]. These sequence elements suggest that the shorter transcript could be synthesized by transcription from a secondary promoter (Fig 2B).

A single transcriptional start site was identified 181 bp upstream of the *walJ* translational start site (Fig 2A). This site is preceded by a perfectly conserved -10 box (TATAAT) and a potential -35 box (TTTAGT). The presence of a TG dinucleotide just upstream from the -10 hexamer indicates that the *walJ* promoter could also belong to the extended -10 promoter family (Fig 2B) [39].

As mentioned above, two potential promoters were identified upstream from the *walRKHI* operon. In order to verify that the shorter cDNA identified by primer extension with the *walR* oligonucleotide corresponds to a *bona fide* transcription initiation site and compare the relative expression levels of the *walRKHI* operon and the *walJ* gene, transcriptional *lacZ* fusions were constructed using the pSA14 vector. DNA fragments were generated by PCR, corresponding to the upstream regions of the *walJ* gene (oligonucleotides OSA479/OSA475; pSA14-P*walJ*) or the *walRKHI* operon, encompassing either both the PI and PII promoters (oligonucleotides OSA477/OSA473; pSA14-P*walR*RI+RII) or the isolated proximal PII promoter region (oligonucleotides OSA478/OSA473; pSA14-P*walR*RII) and cloned between the *Pst*I and *Bam*HI sites of plasmid pSA14. The resulting plasmids were introduced into *S*. *aureus* strain RN4220 and expression was monitored during growth at 37°C. As shown in Fig 2C, the *walR'-lacZ* fusion containing both the PI and PII promoters was strongly expressed (approximately 2500 units mg^-1^ protein; Fig 2C) whereas expression from the *walRKHI* operon PII promoter alone was five-fold lower (approximately 460 Units mg^-1^ protein; Fig 2C), indicating that the shorter cDNA did not result from mRNA processing but is due to transcription from the secondary promoter. The *walJ* promoter *lacZ* fusion was expressed at approximately 540 units mg^-1^ protein whereas the background value for the promoterless pSA14 plasmid alone was 0.65 units mg^-1^ protein (Fig 2C).

Thus, as shown in Fig 2D, the *S*. *aureus wal* locus, unlike that of *B*. *subtilis*, is composed of two distinct σ^A^-dependent transcription units, one encompassing *walRKHI* and a second monocistronic unit corresponding to *walJ*. Furthermore, transcription of the *walRKHI* operon involves dual σ^A^-dependent promoters with different expression levels.

### WalH and WalI are membrane-anchored extracellular proteins

Analysis of the primary structures of the Wal proteins using the Phobius web server (http://phobius.sbc.su.se; [27]) suggests that WalR (233 aa) and WalJ (266 aa) are likely cytoplasmic. In contrast, WalH (444 aa) and WalI (262 aa) have predicted single transmembrane domains (residues 7–27 and 9–26, respectively), whereas WalK (608 aa) has a typical histidine kinase domain structure with two amino-terminal transmembrane domains (residues 12–33 and 183–202) flanking a 148 amino acid extracellular loop. In order to determine the membrane topology of WalH and WalI, we used the *pho-lac* dual reporter system in *E*. *coli* that allows distinction between extracellular (alkaline phosphatase activity), and intracellular protein localization (β-galactosidase activity) [40]. Carboxy-terminal in-frame fusions of the ‘*phoA’-‘lacZ’* reporter gene with DNA fragments encoding the first 40 amino acids of WalJ (fusion point residue E40) and the predicted transmembrane domain coding sequences of WalH (D40) and WalI (N40) were created with the pKTop plasmid [26]. The resulting plasmids were introduced into *E*. *coli* and the strains tested for enzymatic activities on LB plates containing X-Pho as a reporter for alkaline phosphatase activity and Red-Gal for β-galactosidase activity.

**Fig 3:**
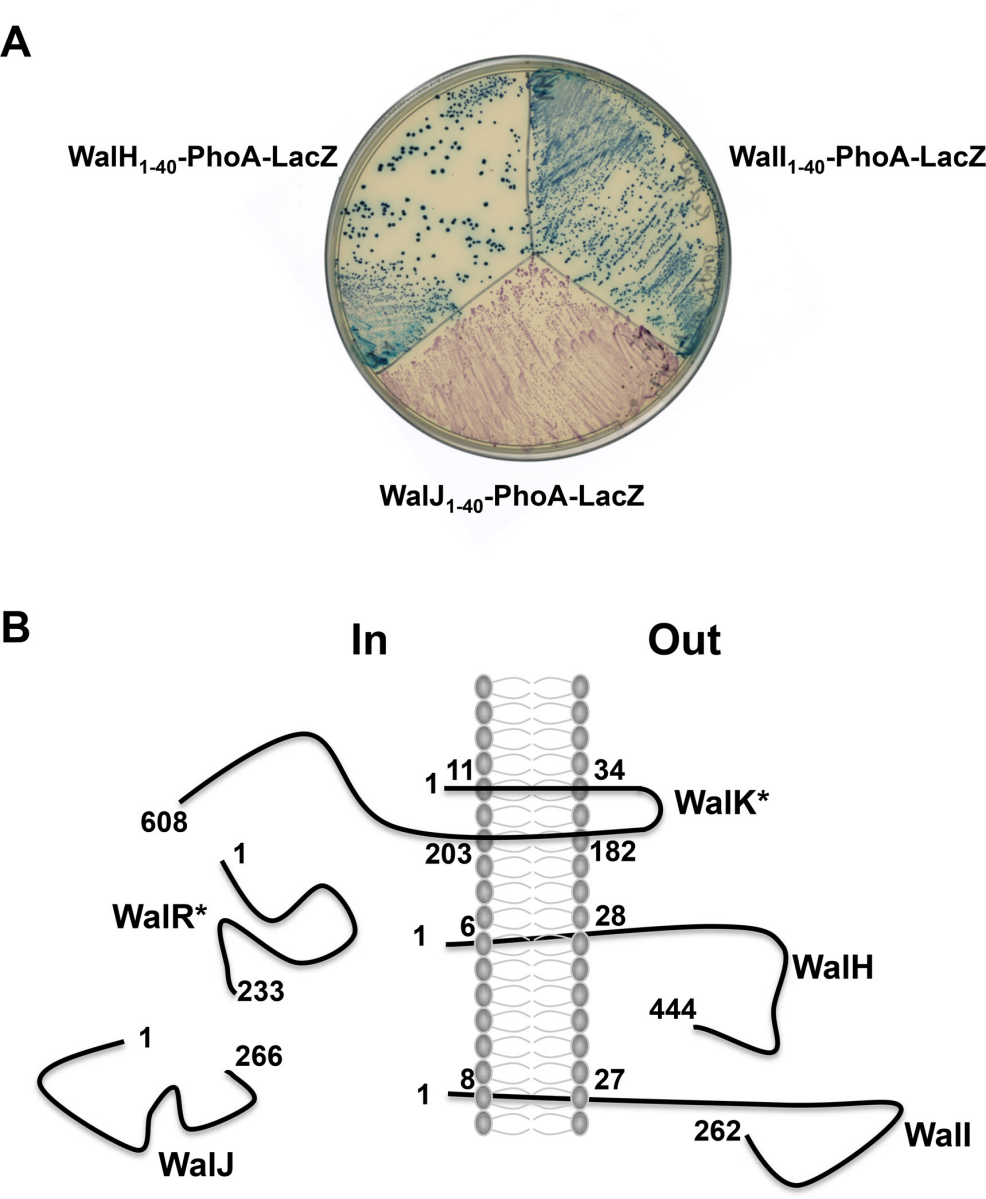
WalH and WalI are membrane-anchored extracellular proteins. (A) *E*. *coli* DH5α cells producing ‘PhoA’-‘LacZ’ fusion proteins (WalH_1-40_-PhoA_21-471_-LacZ_4-60_, WalI_1-40_-PhoA_21-471_-LacZ_4-60_, or WalJ_1-40_-PhoA_21-471_-LacZ_4-60_) were plated on indicator medium with two chromogenic substrates, Red-Gal (for β-galactosidase activity) and X-Pho (for phosphatase activity). Blue coloring of the colonies (high phosphatase activity) indicates a membrane or extracellular localization of the fusion point. Red coloring of the colonies (high β-galactosidase activity) indicates cytosolic localization of the fusion point. (B) Schematic representation of Wal protein localization and topology with respect to the cell membrane. In the case of WalK and WalR, topology and localization were deduced from primary sequence analysis using the Phobius Hidden Markov Model (indicated by stars).

As shown in Fig 3A, strong alkaline phosphatase activity is detected with ‘*phoA’-‘lacZ’* fusions to the first 40 AA of WalH or WalI. These results indicate that WalH and WalI are anchored to the cell membrane through their amino-terminal transmembrane domain, and are consistent with the remainder of the proteins being located extracellularly (residues 28 to 444 for WalH and 27 to 262 for WalI) (Fig 3B). The WalJ_1-40_-‘PhoA’-‘LacZ’ fusion leads to β-galactosidase activity, consistent with the predicted cytosolic localization for WalJ (Fig 3A and 3B).

### WalH interacts with both WalI and WalK

**Fig 4:**
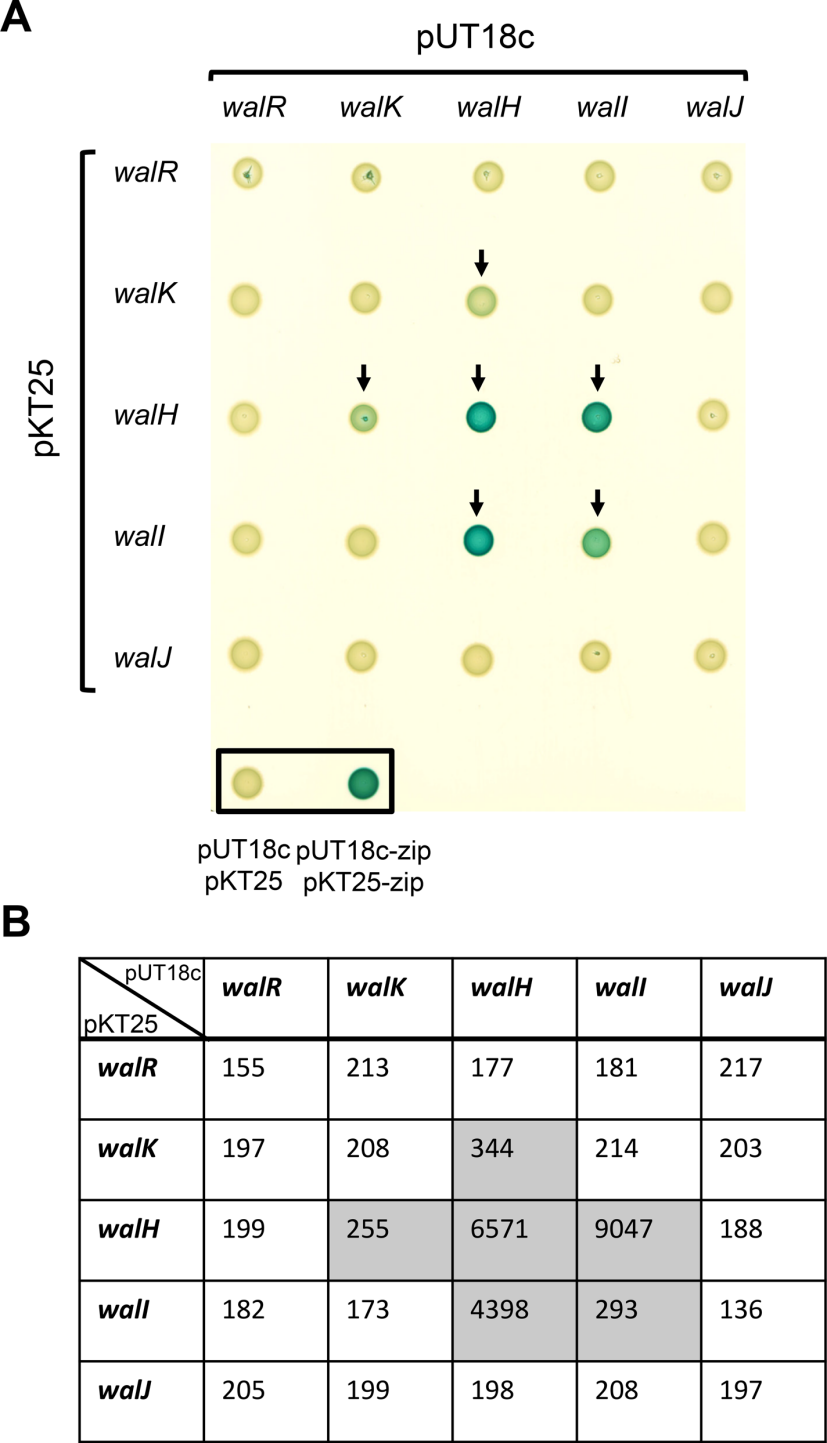
WalH interacts with both WalI and WalK. (A) The five Wal proteins (WalR, WalK, WalH, WalI, WalJ) were systematically tested for pairwise interactions in *E*. *coli* using the BACTH complementation assay by fusing the corresponding full length coding sequences of each of the *wal* genes to those of the T25 or T18 adenylate cyclase domains. DHT1 *E*. *coli* strains co-transformed with each possible combination of plasmids were spotted on LB agar plates with X-Gal as a chromogenic reporter of β-galactosidase activity (see Materials and Methods). Empty pUT18c and pKT25 vectors were also co-transformed in DHT1 as a negative interaction control, and pUT18-zip and pKT25-zip as a strong positive interaction control (boxed spots). Arrows show β-galactosidase positive cells indicating protein-protein interactions. (B) Quantitative β-galactosidase activity assays for each strain shown in panel A (expressed in Miller Units). Shaded cells indicate significant β-galactosidase activities resulting from positive protein-protein interactions.

The conserved gene order of the *wal* locus in Bacilli and the translational coupling of the *walRKHI* operon strongly suggest that the Wal proteins interact. In order to test possible interactions between the Wal proteins, we used the bacterial adenylate cyclase two-hybrid system (BACTH). We fused the full-length WalK, WalR, WalH, WalI and WalJ proteins to the C-terminal domain of either the T18 or T25 subunits of *Bordetella pertussis* adenylate cyclase using the pKT25 and pUT18c plasmids [29]. To probe putative interactions, *E*. *coli* strain DHT1 was co-transformed with combinations of pKT25 and pUT18c derivatives carrying translational fusions to the different *wal* genes. Upon protein-protein interactions, the proximity between the T18 and T25 subunits restores adenylate cyclase activity, leading to cAMP synthesis and activation of the lactose operon. Interactions were tested both by spotting the resulting strains on LB plates containing X-Gal and by measuring β-galactosidase activity (Fig 4A and 4B). To determine pair-wise interactions, we chose a β-galactosidase activity cut-off value of 250 Miller Units to indicate a positive interaction between the protein fusions.

Strong β-galactosidase activity was observed with the pUT18c-*walH*/pKT25-*walH* and pUT18c-*walI*/pKT25-*walI* combinations, indicating that both WalH and WalI self-associate, and the appropriate plasmid pairs allowed us to show that the WalH and WalI proteins also interact with each other (Fig 4A and 4B). Although it is well known that two-component system histidine kinases and response regulators homodimerize, we did not detect self-interaction of WalK or WalR, likely due to instability of the complexes. Concerning interactions between the accessory Wal proteins and the WalKR TCS, the results show that WalH forms a complex with the WalK histidine kinase, but no association between WalI and WalK was detected (Fig 4A and 4B). Thus, the three membrane proteins encoded by the *wal* locus, *i*.*e*. the WalK histidine kinase and the WalH and WalI accessory proteins, associate through interactions with WalH, which appears to be the keystone of the complex. We did not detect any interactions of WalK, WalH or WalI with the cytoplasmic proteins of the system, WalR and WalJ, suggesting either that they do not associate with the other Wal components or that the interaction is transient and that the complex is not sufficiently stable to be detected using this technique.

### WalK and WalH are localized at the division septum

We have shown that WalK, WalH and WalI form a complex. To confirm the results of the BACTH experiments and determine the subcellular localization of this complex in *S*. *aureus* cells, we examined strains expressing translational GFP fusions by fluorescence microscopy. A dedicated plasmid was constructed, pOLSA (see Materials and Methods) allowing translational fusions to GFP. This plasmid is derived from pCN51 in which the P*cad* cadmium-inducible promoter drives expression of the downstream gene [31]. We added an adapter sequence encoding a flexible hexapeptide linker (P-G-S-G-S-G) to avoid steric hindrance between the protein of interest and the fluorescent marker, followed by the GFPopt gene, a codon optimized GFP gene for low-GC% Gram-positive bacteria derived from plasmid pTetONGFPopt [32]. The resulting pOLSA vector allows inducible production of fluorescent protein fusions. Plasmids pOLSA-*walK*, pOLSA-*walH*, pOLSA-*walI* and pOLSA-*walJ* were constructed, fusing the full length coding sequences of the *wal* genes to GFP, and introduced into *S*. *aureus* strain HG001. The resulting strains were grown in TSB until OD_600 nm_ = 0.2, and CdCl_2_ (0.25 μM) was added to the cultures in order to induce expression of the gene fusions. Cells were then harvested at OD_600 nm_ ≈ 1.5 and prepared for fluorescence microscopy (see Materials and Methods).

**Fig 5:**
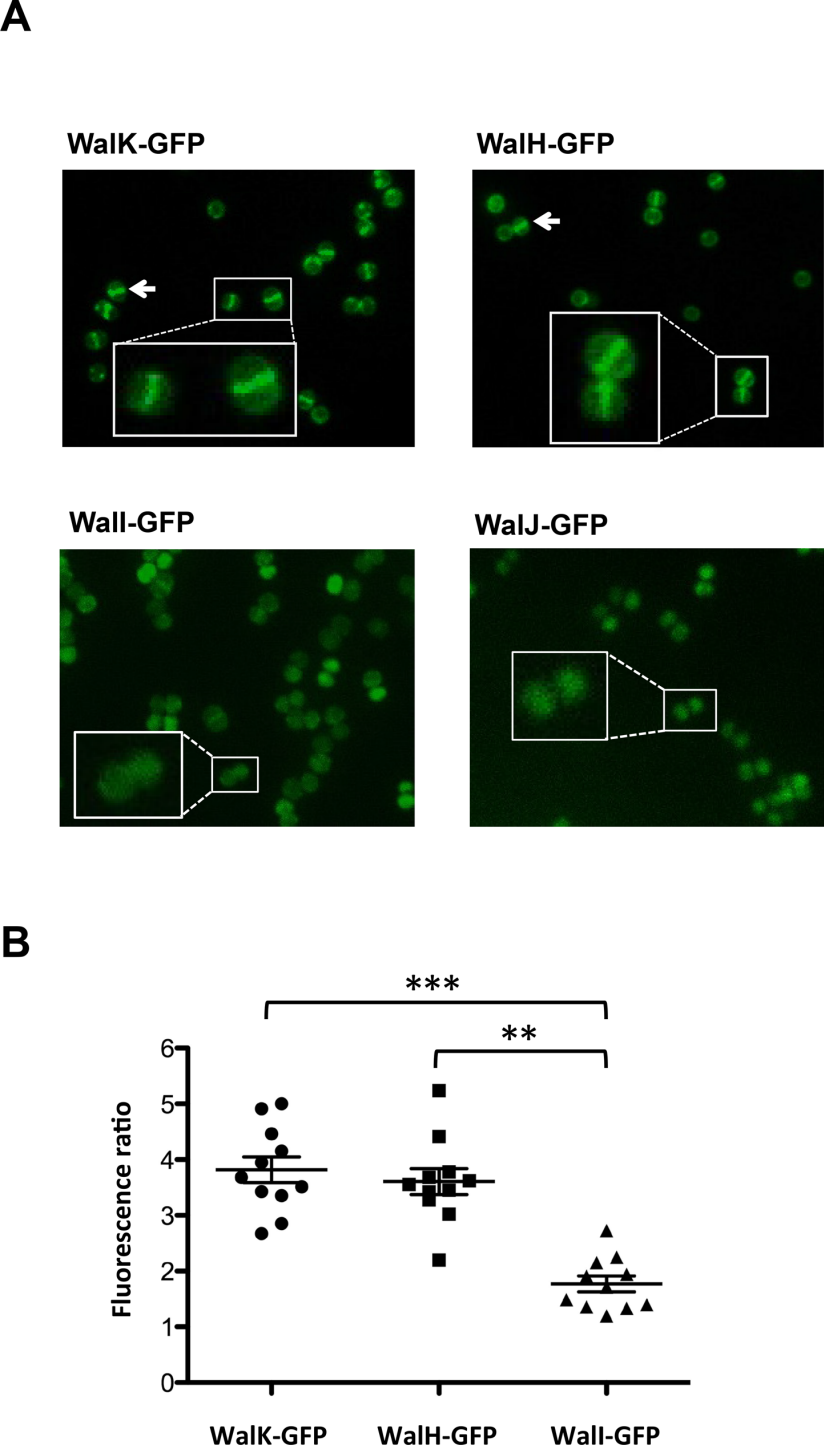
WalK and WalH are localized at the cell division septum. (A) *S*. *aureus* HG001 strains producing fluorescent Wal protein fusions (WalK-GFP, WalH-GFP, WalI-GFP, WalJ-GFP) were grown in TSB and observed in mid-exponential phase by fluorescence microscopy. White arrows and zoomed views indicate septal enrichment. (B) Fluorescence ratios (septum/lateral membrane) were quantified for strains producing membrane protein fusions using ImageJ software and plotted using GraphPad Prism. Horizontal lines correspond to average fluorescence ratios with values greater than 2, indicating preferential septal localization (see Materials and Methods). ** *P*<0.002, *** *P*<0.001 as determined by the Wilcoxon signed-rank test.

As shown in Fig 5A, the localization patterns of WalK and WalH were identical, with a strong concentration of the fusion proteins at the division septa (white arrows and zoomed in views), whereas WalI was found to be evenly distributed in the cell membrane and WalJ was located throughout the cytoplasm, in agreement with the ‘*phoA’-‘lacZ’* analysis.

In order to confirm these observations, fluorescence ratios were calculated by measuring fluorescence at the septum versus that at the lateral membrane. Indeed, since the septum contains two membranes, if a fluorescent protein is homogeneously distributed throughout the cell membrane, the intensity of the fluorescent signal at the septum will be approximately twice that at the lateral membrane, whereas the ratio should be greater than 2 if the protein is specifically accumulated at the division septum. As shown in Fig 5B, fluorescence ratios (septum/lateral membrane) were significantly higher than 2 for both WalK and WalH, confirming that they are preferentially localized at the division septum, whereas WalI was evenly distributed throughout the cell membrane.

### The absence of WalH and/or WalI leads to increased expression of *atlA* and decreased biofilm formation but not to increased autolysis

The *walRK* genes are always followed by *walH*, *walI* and *walJ* in the Bacilli class of bacteria, except for Streptococcaceae, where *walH* and *walI* are absent from the genome and the locus is reduced to *walRKJ* [4]. The strong conservation of the gene order suggests functional interactions between WalH, WalI and WalJ and the WalKR TCS [11]. In order to identify a functional role for WalH, WalI and WalJ, we generated *S*. *aureus* mutant strains lacking either *walH*, *walI*, *walJ* or both *walH* and *walI*. Growth of the resulting mutant strains was identical to that of the parental HG001 strain (S1 Fig), indicating that contrary to the WalKR system, which is essential for cell viability, WalH, WalI and WalJ do not play crucial roles in *S*. *aureus* physiology. Indeed, although the Δ*walH* and Δ*walHI* mutants displayed a slight lag during the first hour post inoculation, the growth rate and OD_600nm_ from then on were not significantly different from those of the other strains including the parental strain (S1 Fig). Doubling times (http://www.doubling-time.com/compute.php) were calculated during the exponential growth phase (56 min to 214 min) and gave identical values of 36 min for each strain. This is quite different from the situation in *Bacillus subtilis* where the absence of either WalH or WalI leads to a cell growth defect [12].

**Fig 6:**
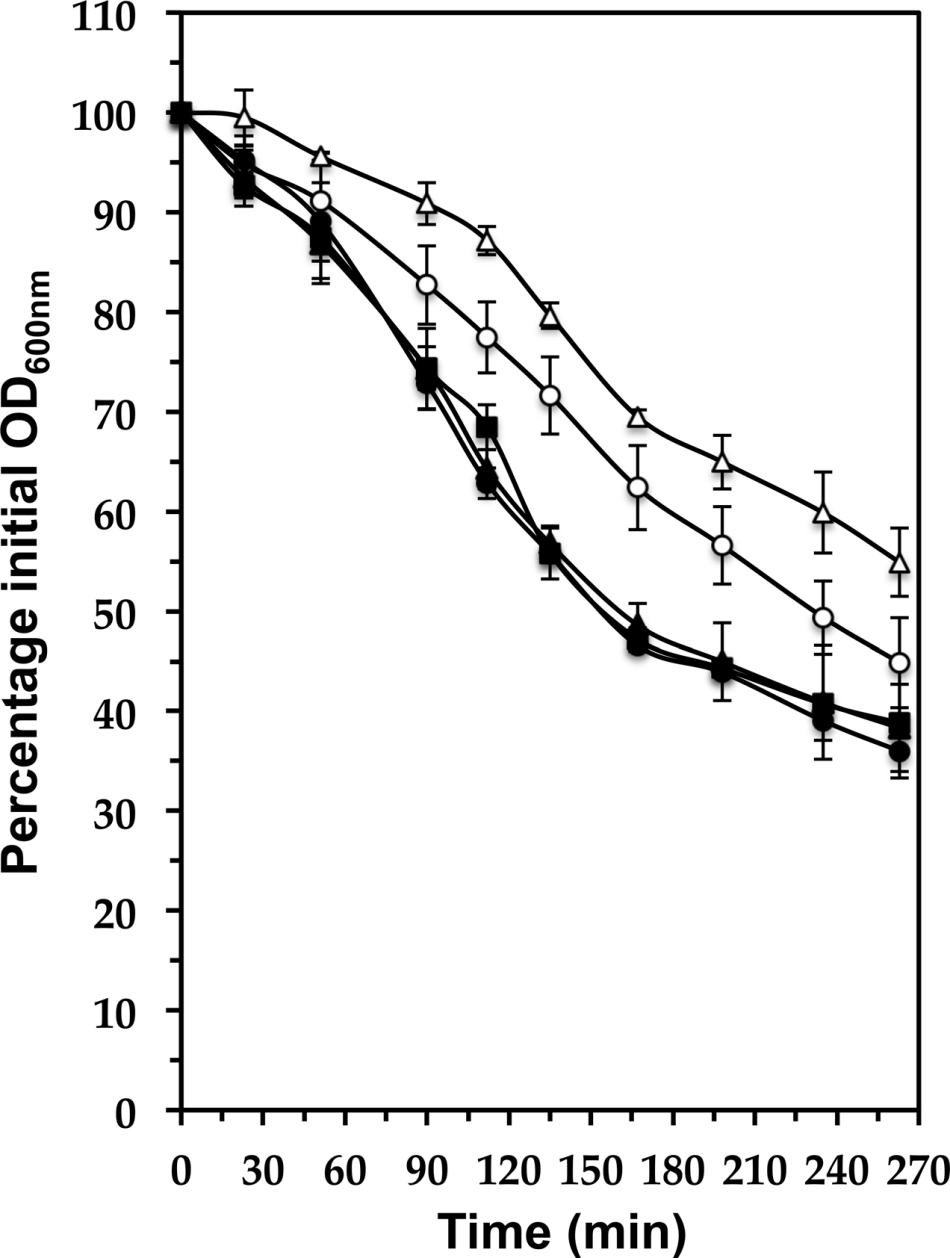
Triton-induced autolysis is decreased in the absence of WalH. Bacteria were grown in TSB at 37°C with shaking until OD_600 nm_ ≈ 1, pelleted (10 min; 5,400 x *g*), resuspended in phosphate buffered saline (PBS) with Triton X-100 (0.1%), and incubated at 37°C with shaking. Lysis was determined as the decrease in OD_600 nm_ over time and indicated as a percentage of the initial OD (measured OD_600 nm_ / initial OD_600 nm_). Results are shown as the mean and standard deviation of three independent experiments. Strains: HG001 (■); ST1397 Δ*walH* (○); ST1410 Δ*walHI* (△); ST1415 Δ*walH* pMK4Pprot-*walHI* (●); ST1417 Δ*walHI* pMK4Pprot-*walHI* (▲).

The Δ*walH* and Δ*walI* mutants of *B*. *subtilis* also display a cell wall defect and increased autolysis, due to increased WalKR activity [12]. We have shown that increased WalR activity in *S*. *aureus* also leads to increased autolysis [6]. To test whether the absence of WalH and/or WalI is associated with increased susceptibility to cell lysis, we tested the effect of a non-ionic detergent, Triton X-100, on autolysis. Triton X-100 is thought to trigger autolysis by removing lipoteichoic acids, which act as inhibitors of endogenous autolysins [41]. Cells were grown in TSB until approximately OD_600nm_ = 1. Cells were then harvested and resuspended in PBS containing 0.1% Triton X-100 (See Materials and Methods). Autolysis rates were measured by following the decrease in OD_600 nm_ over time. Autolysis of the Δ*walH* mutant was found to be slower, as shown in Fig 6. Simultaneous deletion of both *walH* and *walI* led to the same autolysis resistance phenotype as the *walH* deletion, and complementation of the Δ*walH* or Δ*walHI* mutants restored autolysis rates to those of the parental HG001 parental strain (Fig 6). Deletion of *walI* alone had no effect on cell lysis, which was identical to that of the HG001 parental strain under the same conditions (S2 Fig). This contrasts with the situation in *B*. *subtilis* where the Δ*walH* and Δ*walI* mutants display increased autolysis, suggesting that WalKR activity in *S*. *aureus* is not higher in the absence of WalH or WalI.

**Fig 7:**
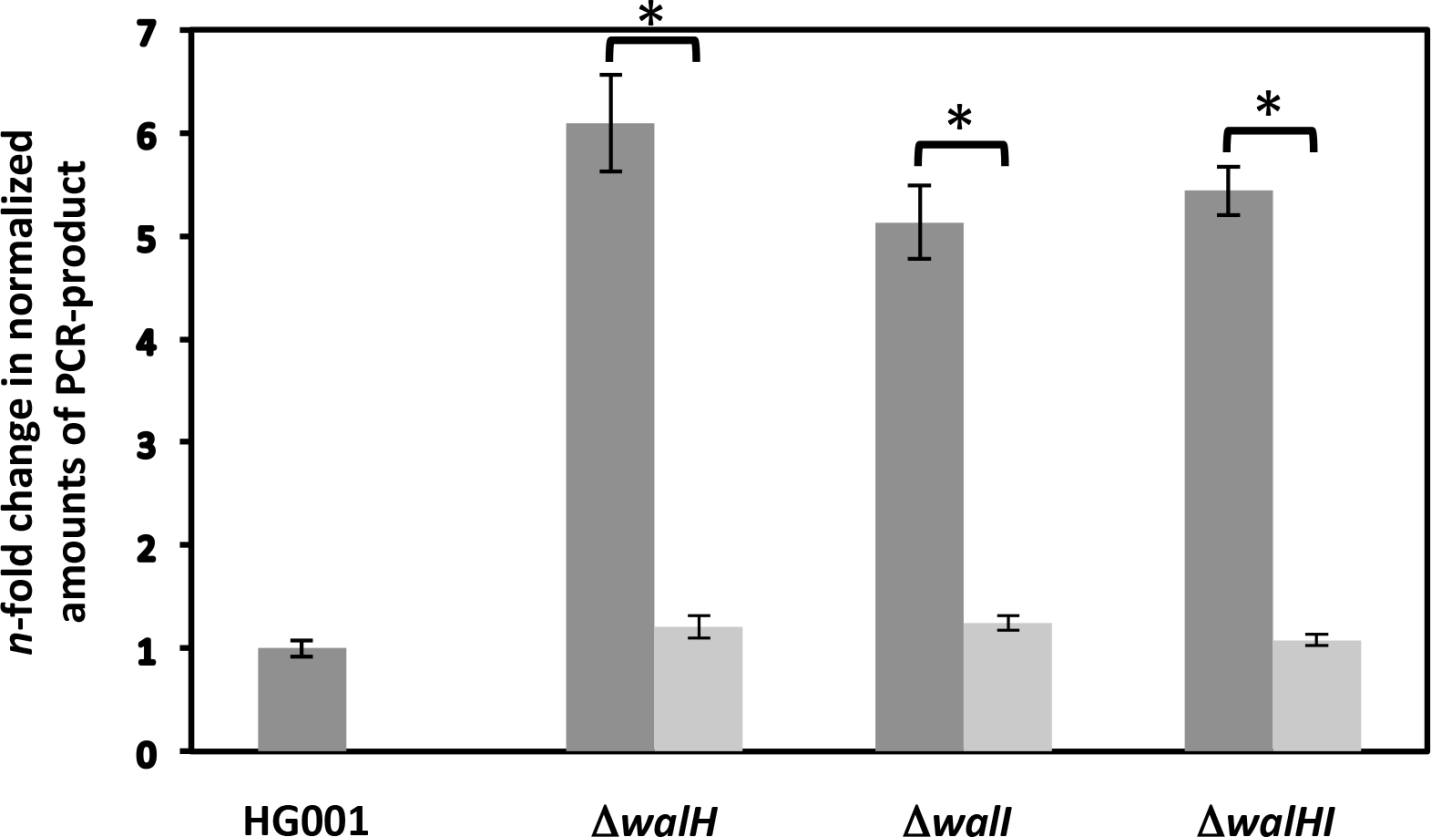
Expression of *atlA* is increased in the Δ*walH*, Δ*walI* and Δ*walHI* mutants. The HG001 parental strain and the Δ*walH*, Δ*walI* and Δ*walHI* mutant strains were grown in TSB rich medium until OD_600 nm_ = 1. Total RNA was extracted and quantitative real time PCR was used to compare gene expression. Expression levels were normalized using 16S rRNA as an internal standard and are indicated as the *n*-fold change with respect to the HG001 parental strain, expressed as means and standard deviations. Dark grey bars indicate expression levels in the parental and mutant strains and light grey bars correspond to values in the complemented strains carrying pMK4-Pprot*walHI*. * *P*<0.05 as determined using Student’s *t*-test.

Studies in *S*. *aureus*, *B*. *subtilis* and *Streptococcus pneumoniae* have shown that cell wall metabolism is the main function regulated by the WalKR system [3, 7–9]. As shown in this work, WalH is a membrane-anchored protein interacting with both WalI and the WalK histidine kinase, leading us to test whether WalH could affect WalKR-dependent regulation of cell wall hydrolase genes. AtlA and Sle1 are the two major autolysins of *S*. *aureus*, playing a crucial role in daughter cell separation by splitting the septum during the division process [42, 43]. We have previously shown that expression of *sle1* and *atlA* is positively controlled by WalKR [3, 6]. The WalKR-activated *saouhsc_00773* gene encodes a protein harboring a LysM cell wall binding domain and a CHAP domain suggesting a role in cell wall degradation [3]. We compared expression of *atlA*, *sle1* and *saouhsc_00773* in the parental HG001 strain and in the Δ*walH*, Δ*walI*, and Δ*walHI* mutant strains by quantitative real time PCR (qRT-PCR). As shown in Fig 7, expression of *atlA* is increased five- to six-fold in the Δ*walH*, Δ*walI* and Δ*walHI* mutants, however expression of *sle1* and *saouhsc_00773* was not significantly different in any of the mutants as compared to the parental strain (S3 Fig). Complementation of the Δ*walH*, Δ*walI* and Δ*walHI* mutants with the pMK4-Pprot*walHI* plasmid fully restored *atlA* expression levels to those of the parental HG001 strain (Fig 7). Since expression of *sle1* and *saouhsc_00773* is strongly regulated by WalKR [3, 6], this suggests that WalH and WalI are not involved in WalKR-dependent regulation of these genes under our conditions, and that their effect on *atlA* expression may be indirect, *ie* that they are not acting by increasing WalKR activity.

In order to verify that the absence of WalH and WalI does not lead to a cell wall defect that could be linked to either increased or decreased WalKR activity, strains HG001 and ST1410 (Δ*walHI*) were grown overnight in TSB and embedded in thin sections for ultrastructure examination by transmission electron microscopy. No significant differences in cell division, septum placement or cell wall thickness were seen between the parental strain and the Δ*walHI* mutant (S4 Fig). We also examined the effect of WalH and WalI on lysostaphin sensitivity. Lysostaphin is a glycyl-glycine endopeptidase that specifically cleaves the pentaglycine cross-bridges of the staphylococcal cell wall, leading to rapid lysis of the bacteria. Strains were grown in TSB until mid-exponential phase (OD_600nm_ = 1). Cells were then pelleted, washed and resuspended in PBS. Lysostaphin-induced lysis was then followed on the nongrowing cells in the presence of lysostaphin (200 ng/ml) at 37°C and measured as the decline in OD_600nm_ over time. No difference in lysostaphin sensitivity was observed in the absence of WalH and/or WalI as compared to the parental HG001 strain (S5 Fig).

**Fig 8:**
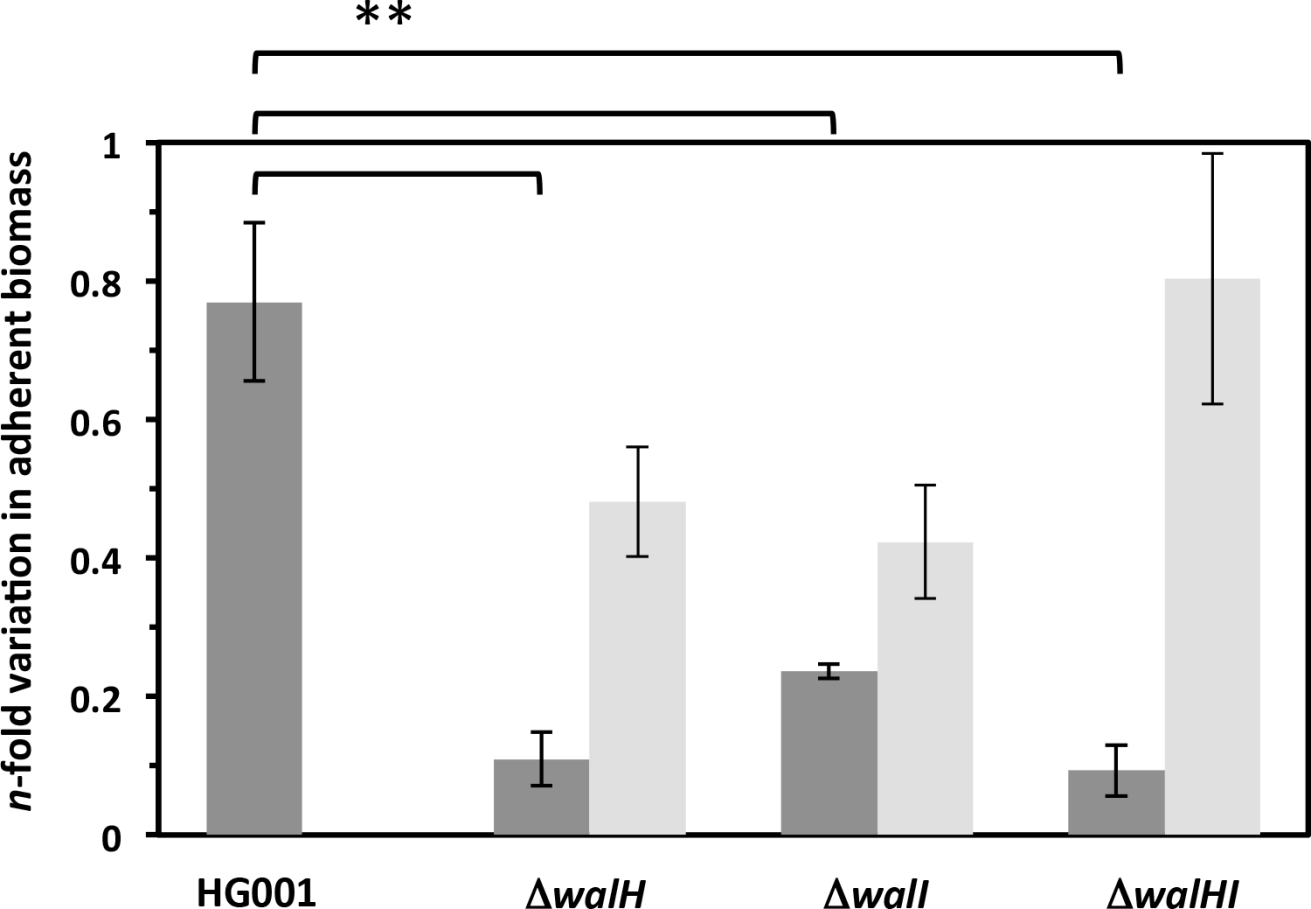
Biofilm formation is decreased in the Δ*walH*, Δ*walI* and Δ*walHI* mutants. Biofilm assays were performed in microtiter plates after growth at 37°C for 24 h. Adherent biomass was quantified, normalized to the OD_600 nm_ of each cell culture and represented as n-fold variation compared to the parental strain. Dark grey bars indicate biomass levels in the parental and mutant strains and light grey bars correspond to values in the complemented strains carrying pMK4-Pprot*walHI*. Experiments were carried out in quadruplicate and standard deviations are indicated. ** *P*<0.01 as determined using Student’s *t*-test.

We previously showed that *S*. *aureus* biofilm formation is enhanced when *walRK* expression is increased or when WalR activity is higher [3, 6]. We therefore tested biofilm formation for the Δ*walH*, Δ*walI* and Δ*walHI* mutants. As shown in Fig 8, biofilm formation was strongly decreased in the absence of WalH and/or WalI. Complementation of the Δ*walH*, Δ*walI* and Δ*walHI* mutants with the pMK4-Pprot*walHI* plasmid restored biofilm formation to levels comparable to those of the parental HG001 strain (Fig 8). This again suggests that WalKR activity is not increased in the Δ*walH*, Δ*walI* and Δ*walHI* mutants, unlike the situation in *B*. *subtilis*.

### Evolution of *wal* operon structure and function among Firmicutes

The unique nature of the *wal* operon structure in *Staphylococcus aureus* prompted us to carry out a phylogenomic analysis of this locus. The *walRK* genes are highly conserved in Bacilli, displaying striking synteny with the *walHIJ* genes in the analyzed genomes, strongly suggesting functional linkage (S1 Table). As previously reported, the *walH* and *walI* genes are absent from the genomes of Streptococcaceae [4, 44], which nevertheless maintain synteny between *walRK* and *walJ*. Interestingly, we observed that *walJ* is missing from all *Leuconostoc* genomes (S1 Table).

**Fig 9:**
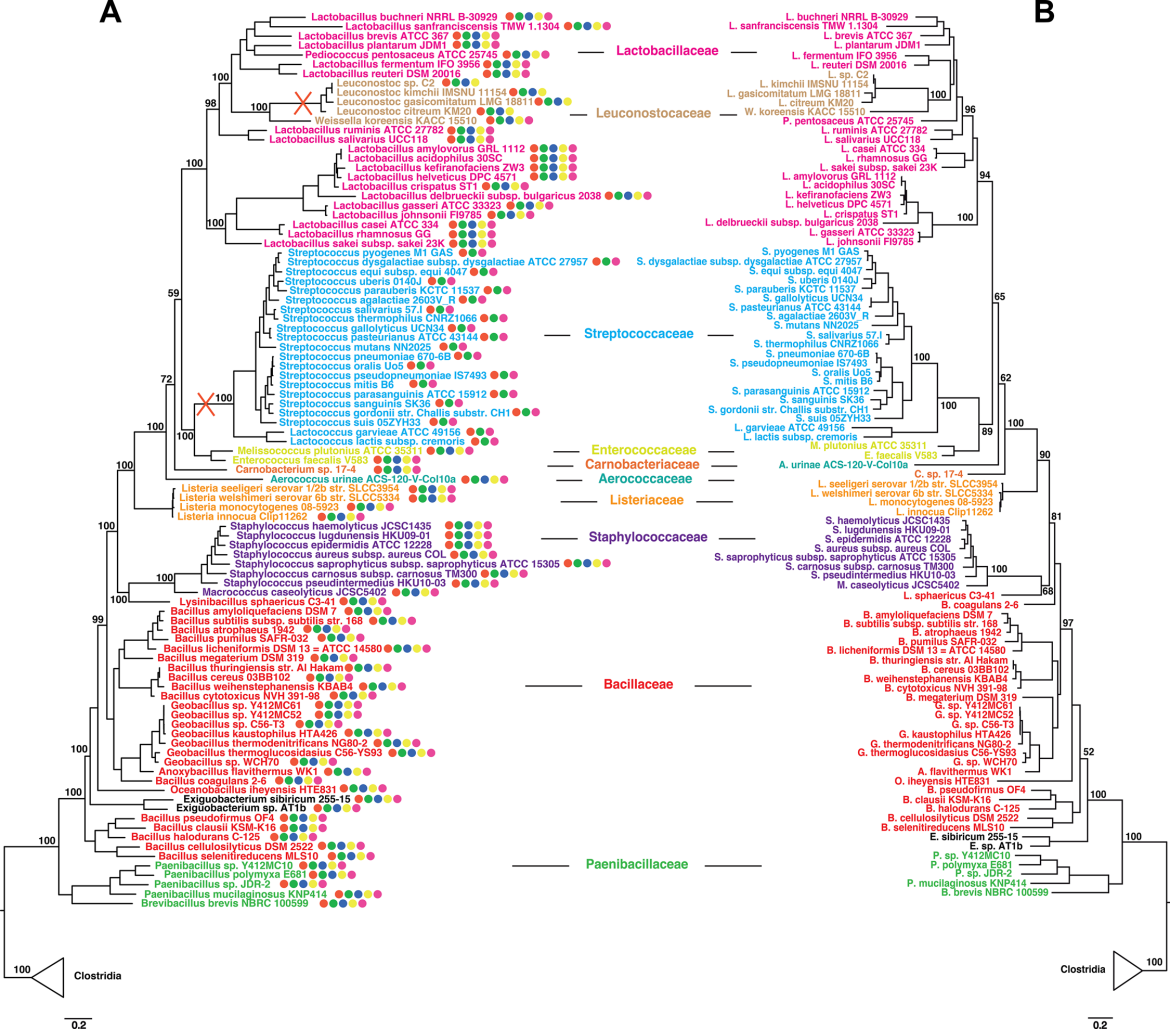
Phylogenetic relationships within the Bacilli class and inferred losses of *wal* genes. (A) Maximum likelihood phylogeny of Bacilli based on a concatenation of 47 ribosomal proteins comprising 5,945 amino acid positions. The conservation of *wal* genes in each genome is indicated by circles: *walR* (red), *walK* (green), *walH* (blue), *walI* (yellow), *walJ* (purple). Red crosses indicate the loss of *walH* and *walI* in Streptococcaceae and of *walJ* in *Leuconostoc*. (B) Maximum likelihood phylogeny based on a concatenation of WalR, WalK, WalH, WalI and WalJ protein sequences comprising 1,102 amino acid positions. For both analyses, values at nodes represent bootstrap proportions calculated on 1,000 resamplings of the original data set. For clarity, only the values corresponding to monophyly of families and their evolutionary relationships are shown. The scale bar represents the average number of substitutions per site. For details on analyses, see Materials and Methods.

We sought to understand if this conservation is due to reshuffling through horizontal gene transfer among Bacilli or a consequence of vertical inheritance. We therefore carried out a phylogenomic analysis of the 5 gene *wal* locus in 119 representatives of the Bacilli class plus 24 representatives of the Clostridia class as an outgroup. We built a reference species tree by concatenation of 47 widely distributed ribosomal proteins (Fig 9A, see Materials and Methods for details). In parallel, we analyzed the phylogenies of all 5 Wal proteins. Albeit generally poorly resolved, due to the well known lack of a strong phylogenetic signal in single marker genes, these Wal trees were globally consistent with the reference species tree, allowing them to be concatenated into a single character supermatrix of over a thousand positions. The resulting tree (Fig 9B) is highly consistent with the reference species tree, indicating that the ancestor of Bacilli already harbored a 5 gene *wal* cluster, and that this system was inherited in a largely vertical fashion through speciation events (mapped on Fig 9A). This is unusual for gene clusters, which should be easy to exchange among bacteria. Either the essential nature of the Wal system is such that all attempts at homologous replacement are strongly counterselected, or the system works with additional components that lie elsewhere in the genomes, preventing transfer of a fully functional system. Moreover, our analysis unequivocally shows that the lack of some *wal* genes in specific genomes is due to gene loss. This is the case for Streptococcaceae, where *walH* and *walI* appear to have been neatly excised from the cluster, as well as for *Leuconostoc* species where *walJ* was lost (indicated by red crosses in Fig 9A).

Streptococcaceae differ from other Firmicutes with respect to the WalKR regulatory pathway. Indeed, whereas the genes encoding the WalR response regulator (RR) and the WalK histidine kinase (HK) are both essential for cell viability in other Firmicutes, only the *walR* gene is essential in Streptococcaceae, suggesting that the RR can be phosphorylated either by a non-cognate HK or by small phosphate donors such as acetyl phosphate. Non-essentiality of *walK* in Streptococcaceae is always associated with the absence of *walH* and *walI*. Although Streptococci and Staphylococci differ in physiology, this observation prompted us to test whether *walK* was still essential in a *S*. *aureus* strain that lacks both *walH* and *walI*. We used a deletion strategy with the pMAD allelic replacement vector [19]. The region upstream from *walK* was fused to the region downstream of *walI* in the plasmid construct, pMAD*walK* (S6 Fig). When cells are grown at the non-permissive temperature for pMAD replication, integration of the entire circular plasmid occurs by a Campbell-type single crossover event, with concomitant duplication of the *walR* and *walJ* genes (S6 Fig). Depending on the recombination site, this could then result either in a separation of *walRK* from their upstream promoter, with one copy of *walR* alone expressed from its promoter (S6 Fig, event A), or conservation of the *walRK* locus transcribed from its own promoter (S6 Fig, event B). We determined the integration site for 8 clones, showing that event B, which leaves transcription of *walRK* intact, occurred in 100% of the integrants. Growth temperature shifts were then performed in order to favor plasmid excision (second crossover event). Depending on the recombination regions, this second crossover could result either in deletion of the *walK* gene (S6 Fig, event C) or conservation of the *wal* locus structure in the original Δ*walHI* recipient strain (S6 Fig, event D). Two independent experiments were performed and among the 107 tested clones, all retained the *walK* gene. These results strongly suggest that in *S*. *aureus*, the *walK* gene remains essential, even in the absence of WalH and WalI. They also suggest a global evolution of the WalKR system in Streptococcaceae that has led to non-essentiality of the *walK* gene.

## Discussion

Two-component systems have evolved in free-living Eubacteria as highly conserved mechanisms for adaptation to environmental changes. Among these, the WalKR TCS, ubiquitous among low G+C% bacteria and specific to the Firmicutes phylum, is one of the few that have been shown to be essential for cell viability. This has been linked to its major role in controlling cell wall hydrolases, both in *S*. *pneumoniae* and *S*. *aureus* [9, 10]. Studies of the WalKR system have essentially focused on the *walR* and *walK* genes, yet they are part of a conserved locus encompassing five genes (*walR*, *walK*, *walH*, *walI*, and *walJ*) except in Streptococcaceae, which lack the *walH* and *walI* genes and have a tricistronic *walRKJ* operon [4, 44, 45]. In *B*. *subtilis*, the *wal* locus contains an additional gene, *yycK* (aka *yyxA*), located downstream from *walJ* and encoding an HtrA protease orthologue, and it has been shown that *walR*, *K*, *H*, *I*, *J* and *yycK* are all co-transcribed [46]. However, YycK does not appear to play a role in WalKR-dependent regulation [47]. The *B*. *anthracis* and *Listeria monocytogenes wal* loci are also followed by a gene encoding a HtrA-like serine protease [4]. In *L*. *monocytogenes*, expression of *htrA* is controlled by the LisK/LisR TCS, and HtrA plays a role in stress response and pathogenesis [48, 49].

The co-occurence and synteny of the *wal* genes is a strong indication of a functional link between the Wal proteins. Studies in *B*. *subtilis* have shown that WalH and WalI interact with the WalK histidine kinase to negatively control its activity [12, 13, 47, 50]. Although no direct links between WalJ and the WalKR two-component system have been clearly demonstrated, WalJ has also been suggested to be involved in regulation of cell wall metabolism. Indeed, in *B*. *subtilis* and *S*. *pneumoniae*, deletion of *walJ* leads to increased sensitivity to some cephalosporin antibiotics and *walJ* is essential in both bacteria when *walRK* expression is reduced [9, 14].

WalK, WalR and WalJ are well conserved in Firmicutes (46%, 75% and 59% amino acid sequence identity between *B*. *subtilis* and *S*. *aureus*, respectively). In contrast, WalH and WalI share no significant similarities, either between different Firmicutes (17% and 21% amino acid sequence identity between *B*. *subtilis* and *S*. *aureus*, respectively) or with each other (16% identity in *S*. *aureus*). Despite their poor conservation, the crystal structures of WalH and WalI from *B*. *subtilis* are remarkably similar, with a common fold [51, 52].

In *S*. *aureus*, nothing was known about the *walH*, *walI* and *walJ* genes. We carried out a detailed analysis of *wal* locus transcription and showed that the first gene of the locus, *walR*, is preceded by two independent σ^A^-type promoters driving expression of *walR*, *walK*, *walH* and *walI*. We note that these experimentally determined promoters are different from the potential promoter previously proposed by *in silico* analysis [53]. A recent study also identified two promoters upstream from the *S*. *aureus walRK* genes, however their position is slightly different from the ones identified here, due to the technique that was used [54]. In that study, a mutation affecting the -10 of the PII promoter identified here led to lowered transcription, in agreement with our results. Based on our established consensus sequence [2], no WalR-binding sites could be identified in the *walRKHI* operon promoter region, consistent with our previous results indicating that expression of the *walRK* genes is not autoregulated [6]. As shown in Fig 2C, the fact that transcription of *walRKHI* occurs from two promoters with different strengths could suggest that one is involved in maintaining a basal level of expression whereas transcription from the other may be regulated in response to specific conditions.

Interestingly, *walJ* was found to be independently transcribed in *S*. *aureus*, whereas in *B*. *subtilis* and *S*. *pneumoniae*, *walJ* is co-transcribed with the preceding *wal* genes [46, 55]. In *S*. *aureus*, the intergenic region between *walI* and *walJ* is unusually large, with a conserved length of 389 bp. A careful survey of the *walI walJ* region in Firmicutes indicates that this large intergenic sequence is particularly well conserved and specific to *S*. *aureus* strains. The *walJ* transcription start site is located 182 bp upstream from the start of the *walJ* coding sequence. This transcribed but untranslated region is highly structured (Pascale Romby, CNRS Strasbourg, personal communication), suggesting it could be involved in post-transcriptional regulation of *walJ* expression. A transcript corresponding to the *walJ* upstream sequence, Teg107, was previously identified by high-throughput sequencing of the *S*. *aureus* transcriptome [56] and our results show that it corresponds to the *walJ* 5'-UTR region. In agreement with our findings, an *in silico* study also predicted that *walJ* is transcribed independently in *S*. *aureus* [57]. Interestingly, *walJ* has been lost in *Leuconostoc* species (Fig 9), where the gene immediately following the *walRKHI* operon encodes an HtrA-like protease, as in *B*. *subtilis* and *L*. *monocytogenes*.

In an effort to understand the roles of the WalH, WalI and WalJ accessory proteins, we constructed Δ*walH*, Δ*walI*, Δ*walHI* and Δ*walJ* mutant strains in *S*. *aureus*. The mutants did not display any distinguishing phenotypes, and cell morphology and growth were not affected. Although a number of reports link the *S*. *aureus* WalKR system and WalH with vancomycin-intermediate resistance (VISA phenotype) [54, 58–63], the Δ*walH*, Δ*walI*, Δ*walHI* and Δ*walJ* mutants were not significantly affected in sensitivity to vancomycin. Indeed, only a very small change was seen for the Δ*walH* and Δ*walHI* strains (MIC increase from 3 μg/ml to 4 μg/ml) which could be fully complemented (S7 Fig), whereas no difference was observed for the Δ*walI* and Δ*walJ* mutants. Furthermore, it was recently reported that *walR* overexpression did not affect vancomycin MICs in *Bacillus anthracis* [64].

Deletion of *walJ* in other bacteria leads to decreased resistance to oxidative stress and cell wall targeting antibiotics, and a DNA partition default during cell division, and more recently was associated with a spontaneous mutator phenotype in *B*. *anthracis* [14–18]. We tested several of these phenotypes with the Δ*walJ* mutant of *S*. *aureus* and saw no difference in chromosomal segregation during bacterial division (S8 Fig) and in sensitivity to paraquat-induced oxidative stress (S9 Fig) or cell wall targeting antibiotics (fosfomycin, penicillin, cefotaxime, cefalotin) (data not shown).

In *B*. *subtilis* the WalK kinase is localized at the division septum in an FtsZ-dependent manner, interacting with the DivIB, Pbp2B and FtsL division proteins, whereas the WalH and WalI proteins are not localized at the septum but around the cell [50, 65]. In non-dividing *B*. *subtilis* cells, in the absence of the divisome, WalH and WalI associate with WalK through their transmembrane helices to inhibit its activity [12, 13, 47]. Thus, in the *B*. *subtilis* model, WalK is localized at the division septum in actively dividing cells, where it can no longer interact with WalH and WalI, and the active WalKR system can then coordinate cell wall plasticity with cell division [4, 50].

Our study has highlighted several major differences between the WalKR systems of *S*. *aureus* and *B*. *subtilis*, with features that appear to be specific to Staphylococci. We show that WalH and WalI are membrane-anchored extracellular proteins, that WalH and WalK interact using the BACTH system in *E*. *coli*, and that in contrast to the situation in *B*. *subtilis*, WalH and WalK are both localized at the division septum. We also showed that WalH and WalI interact with each other, suggesting that WalH, WalI and WalK form a ternary complex. However, although we could not detect any interactions between WalI and WalK, WalI is also found at the septum, even if it does not localize there preferentially, such that all three proteins can interact there. However, we cannot exclude that fusing GFP to WalI may prevent it from localizing efficiently to the septum. We have also shown that the *walJ* gene is transcribed independently from the other *wal* genes in *S*. *aureus*, unlike the *wal* operon of *B*. *subtilis*.

Furthermore, unlike the situation in *B*. *subtilis*, no increased autolysis was observed for the Δ*walH*, Δ*walI* and Δ*walHI* mutant strains (see Fig 6 and S2 Fig), nor did the mutants present growth or cell wall defects (S1 and S4 Figs). On the contrary, Triton X-100 induced autolysis was actually decreased in the Δ*walH* and Δ*walHI* mutant strains, and this phenotype could be fully reversed upon complementation (Fig 6).

Biofilm formation, which we have shown to be directly correlated with WalR activity, was lowered in the Δ*walH*, Δ*walI*, Δ*walHI* mutant strains (Fig 8), and WalKR-dependent expression of *sle1* and *saouhsc_00773* was not affected by the absence of WalH and WalI (S3 Fig). Taken together, these results indicate that WalH and WalI do not negatively regulate WalKR activity in *S*. *aureus* as they do in *B*. *subtilis*, consistent with their divergent sequences. In agreement with our results, a report where full length WalH, WalI, WalK and WalR proteins of *S*. *aureus* were purified and combined *in vitro* showed that WalH and WalI had no significant effect on WalK autophosphorylation or phosphotransfer to WalR [66]. A two-fold increase in autolysis and biofilm formation was recently reported for a *walI* mutant of *S*. *aureus*, however the mutant was a gene disruption, leaving much of the protein produced, and the strain used was a clinical isolate carrying uncharacterized mutations leading to increased biofilm formation and Agr activity [67].

Both *walH* and *walI* have been lost from Streptococcaceae as shown by our phylogenomic analyses (Fig 9). This was suggested to be linked to a lack of capacity for electron transport [44]. However, we saw no difference in expression of *walK*, *walR* or *atlA* during aerobic or anaerobic growth of *S*. *aureus* indicating neither expression levels nor activity of the system are affected under these conditions (S10 Fig). WalH and WalI are absent in Streptococcaceae where the WalK kinase lacks an extracytoplasmic domain and is not essential [4, 45]. By measuring resolution frequencies for tandem chromosomal duplications following integration of plasmids designed to delete *walK*, we showed that WalK remains essential in *S*. *aureus* even in the absence of WalH and WalI (S6 Fig). This is consistent with the fact that in *S*. *pneumoniae*, where WalK is not essential, the WalR response regulator can be phosphorylated by small molecule phosphodonors such as acetyl phosphate [68], whereas purified WalR of *B*. *subtilis* or *S*. *aureus* could not be phosphorylated using radiolabeled acetyl phosphate (S. Dubrac, unpublished results). A recent report has shown that in *Listeria monocytogenes*, WalK also appears as not essential for cell viability, as in Streptococcaceae [69]. However, sequence analysis of the *wal* locus in *Listeria monocytogenes* reveals a very different structure from that of *S*. *aureus* or *B*. *subtilis*: the *walR* gene in *L*. *monocytogenes* does not appear to be co-transcribed with the remaining *walKHIJ* genes. Indeed, the intergenic region between *walR* and *walK* is 184 bp versus 12 in *S*. *aureus*, with a likely Rho-independent transcription terminator located downstream from *walR*. This suggests that WalR in *L*. *monocytogenes* may have evolved so as to be active even in the absence of its cognate kinase, much like in *S*. *pneumoniae*.

Cell wall biosynthesis and degradation require fine-tuning in order to ensure proper cell division and avoid cell lysis. In *B*. *subtilis* WalH and WalI control WalKR activity whereas they are absent in Streptococcaceae. Our results are consistent with the idea that the WalKR system of Staphylococci may represent an evolutionary intermediate between those of *Bacillus* and Streptococci, with a progressive loss of importance of WalH and WalI. In Staphylococci, WalH and WalI are still present, but no longer play an essential role in negatively controlling WalKR activity as in *B*. *subtilis*. This suggests that other components could be involved in fine-tuning WalKR activity, and this hypothesis is currently being investigated in our laboratory.

## Supporting Information

S1 Fig Growth curves of *S*. *aureus* HG001 and *wal* mutant strains. Bacterial cultures were grown overnight, inoculated in TSB at a calculated OD_600nm_ of 0.05 and incubated at 37°C with shaking. Optical densities were followed over a 7.5 hour period. Results are shown as the mean and standard deviation of three independent growth curves. Doubling times (http://www.doubling-time.com/compute.php) were calculated during the exponential growth phase (56 min to 214 min) and gave identical values of 36 min for each strain. Strains: HG001 (■); ST1397 Δ*walH* (◆); ST1130 Δ*walI* (▲); ST1410 Δ*walHI* (△); ST1131 Δ*walJ* (○).
(TIF)

S2 Fig WalI does not affect Triton-induced autolysis. Bacteria were grown in TSB at 37°C with shaking until OD_600 nm_ ≈ 1, pelleted (10 min; 5,400 x *g*), resuspended in phosphate buffered saline (PBS) with Triton X-100 (0.1%), and incubated at 37°C with shaking. Lysis was determined as the decrease in OD_600 nm_ over time and indicated as a percentage of the initial OD (measured OD_600 nm_ / initial OD_600 nm_). Results are shown as the mean and standard deviation of three independent experiments. Strains: HG001 (■); ST1130 Δ*walI* (▲).
(TIF)

S3 Fig WalH and WalI have no effect on expression of the *sle1* and *saouhsc_00773* WalKR regulon autolysin genes. The HG001 parental strain and the Δ*walH*, Δ*walI* and Δ*walHI* mutant strains were grown in TSB rich medium until OD_600 nm_ = 1. Total RNA was extracted and quantitative real time PCR was used to compare gene expression in the strains. Expression levels were normalized using 16S rRNA as an internal standard and are indicated as the *n*-fold change with respect to the parental HG001 strain, expressed as means and standard deviations.
(TIF)

S4 Fig The Δ*walHI* mutant does not display any cell wall defects. Strains HG001 and ST1410 (Δ*walHI*) were grown overnight in TSB and embedded in thin sections for ultrastructure examination by transmission electron microscopy. Bacteria were fixed with 2,5% glutaraldehyde for 2 hrs at RT, postfixed with 1% osmium tetroxide for 1 hr at RT, dehydrated in a graded series of ethanol baths, and embedded in Epon. Thin sections were cut with a Leica Ultramicrotome Reichert Ultracut S, and stained with uranyl acetate and lead citrate. Images were taken with a FEI Tecnai Bio-Twin Transmission Electron Microscope at 120kV. Bars represent 0.5 μm.
(TIF)

S5 Fig Lysostaphin sensitivity of *wal* mutant strains. Bacteria were grown in TSB at 37°C with shaking until mid-exponential phase (OD_600 nm_ ≈ 1). Cells were then pelleted, washed, and resuspended in PBS. Lysostaphin-induced lysis was then followed on the nongrowing cells in the presence of lysostaphin (200 ng/ml) at 37°C, measured as the decline in OD_600nm_ over time and indicated as a percentage of the initial OD (measured OD_600 nm_ / initial OD_600 nm_). Results are shown as the mean and standard deviation of three independent experiments. Strains: HG001 (▰); ST1397 Δ*walH* (●); ST1130 Δ*walI* (▲); ST1410 Δ*walHI* (◆).
(TIF)

S6 Fig The *walK* gene remains essential in *S*. *aureus*, even in the absence of *walH* and *walI*. Schematic representation of the *wal* locus in strain HG001 and the Δ*walHI* mutant and the recombinant pMAD*walK* plasmid designed to delete the *walK* gene in the *walHI* mutant (not to scale). A, B, C, and D indicate the possible recombination events and their frequencies as tested by PCR screening with appropriate primers.
(TIF)

S7 Fig Vancomycin MICs are not significantly affected by the Δ*walH*, Δ*walI*, Δ*walHI* and Δ*walJ* mutations. Bacterial strains were grown overnight at 37°C with shaking in TSB (supplemented with 10 μg/ml chloramphenicol for the complemented strains). Overnight cultures were diluted in TSB to reach an optical density around 0.1 (0.5 McFarland) and flooded on BHI agar plates. The E-tests (Biomérieux) were then applied on dried plates and incubated for 24 hours at 37°C before observation.
(TIF)

S8 Fig Chromosome segregation is not affected in the *S*. *aureus* Δ*walJ* mutant. The HG001 and ST1131 (Δ*walJ*) strains were grown in TSB until OD_600nm_ ≈1. Half of each culture was treated with novobiocin (0.5 mg/ml) in order to inhibit chromosomal replication (this has been shown to amplify the DNA segregation phenotype of a Δ*walJ* mutant strain in *B*. *subtilis*), and incubation was pursued. Cells were harvested after one hour and resuspended in PBS. Cell membranes were labeled with FM1-43FX (red) and DNA with DAPI (blue). Suspensions were immediately mounted with Vectashield and observed with a Nikon Eclipse E600 microscope. Images were acquired with a Nikon Digital Camera DXM1200F. No differences were observed between cultures with or without novobiocin treatment, and the images shown are from the cultures treated with novobiocin. Panels A: membrane labeling (FM1-43FX), B: DNA labeling (DAPI), C: merged. No defects in DNA segregation were observed for the Δ*walJ* mutant as compared to the HG001 parental strain.
(TIF)

S9 Fig The Δ*walJ* mutant strain does not show increased sensitivity to paraquat-induced oxidative stress. *S*. *aureus* strains HG001 and ST1131 (Δ*walJ*) were grown overnight, and inoculated at a calculated OD_600nm_ of 0.05 in TSB supplemented with the indicated concentrations of paraquat (methyl viologen hydrate). Growth was followed by measuring OD_600nm_ over a six hour period. Growth of the HG001 parental strain (closed symbols) and the Δ*walJ* strain (open symbols) was not affected by paraquat concentrations up to 10 mM, whereas growth of both strains was strongly inhibited in the presence of 20 mM paraquat. (circles: 5 mM paraquat; squares: 10 mM; diamonds: 20 mM).
(TIF)

S10 Fig Expression of *walRK* and the *atlA* WalKR-regulated autolysin gene is not affected by anaerobiosis. *S*. *aureus* strain HG001 wild type strain was grown overnight, inoculated in TSB at a calculated OD_600nm_ of 0.05 and half of the culture was incubated at 37°C under agitation whereas the other half was incubated at 37°C in an anaerobic jar. At an OD_600nm_ ≈ 1, cells were harvested and treated for RNA extraction, cDNA synthesis and qRT-PCR analysis (see Materials and Methods section). Data are represented as *n*-fold change during growth under anaerobiosis compared to aerobiosis, expressed as means and standard deviations.
(TIF)

S1 Table Sequence accession numbers for Wal proteins used in the phylogenomic analysis. (PDF)
