# Supplementary figures and images for: Transcriptional Analysis and Subcellular Protein Localization Reveal Specific Features of the Essential WalKR System in Staphylococcus aureus
Source: PLoS One. 2016 Mar 21;11(3):e0151449. doi: 10.1371/journal.pone.0151449 (PMC4801191; doi:10.1371/journal.pone.0151449)

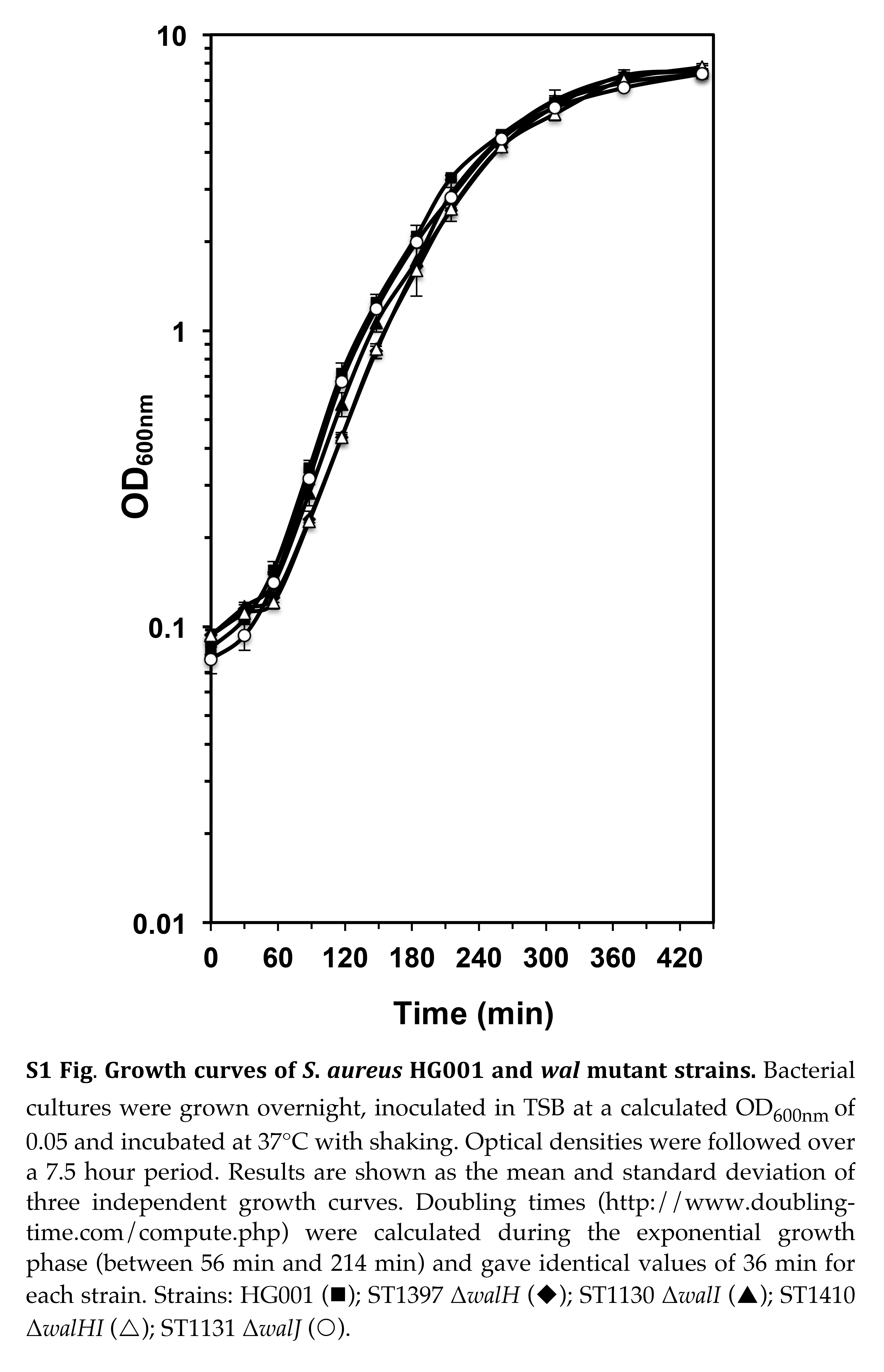

Supplement: S1 Fig — Bacterial cultures were grown overnight, inoculated in TSB at a calculated OD600nm of 0.05 and incubated at 37°C with shaking. Optical densities were followed over a 7.5 hour period. Results are shown as the mean and standard deviation of three independent growth curves. Doubling times (http://www.doubling-time.com/compute.php) were calculated during the exponential growth phase (56 min to 214 min) and gave identical values of 36 min for each strain. Strains: HG001 (■); ST1397 ΔwalH (◆); ST1130 ΔwalI (▲); ST1410 ΔwalHI (△); ST1131 ΔwalJ (○). (TIF) [file pone.0151449.s001.tif]

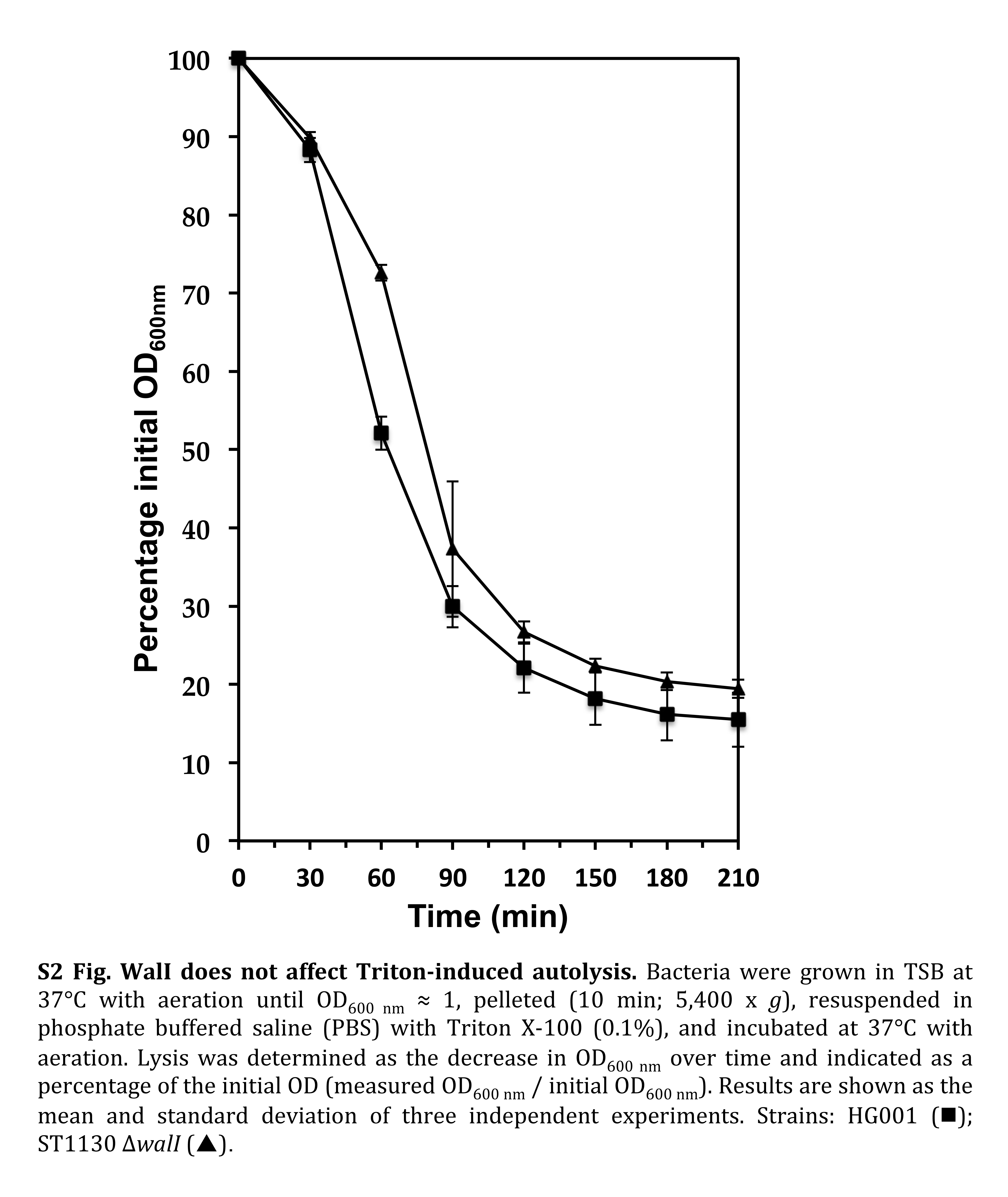

Supplement: S2 Fig — Bacteria were grown in TSB at 37°C with shaking until OD600 nm ≈ 1, pelleted (10 min; 5,400 x g), resuspended in phosphate buffered saline (PBS) with Triton X-100 (0.1%), and incubated at 37°C with shaking. Lysis was determined as the decrease in OD600 nm over time and indicated as a percentage of the initial OD (measured OD600 nm / initial OD600 nm). Results are shown as the mean and standard deviation of three independent experiments. Strains: HG001 (■); ST1130 ΔwalI (▲). (TIF) [file pone.0151449.s002.tif]

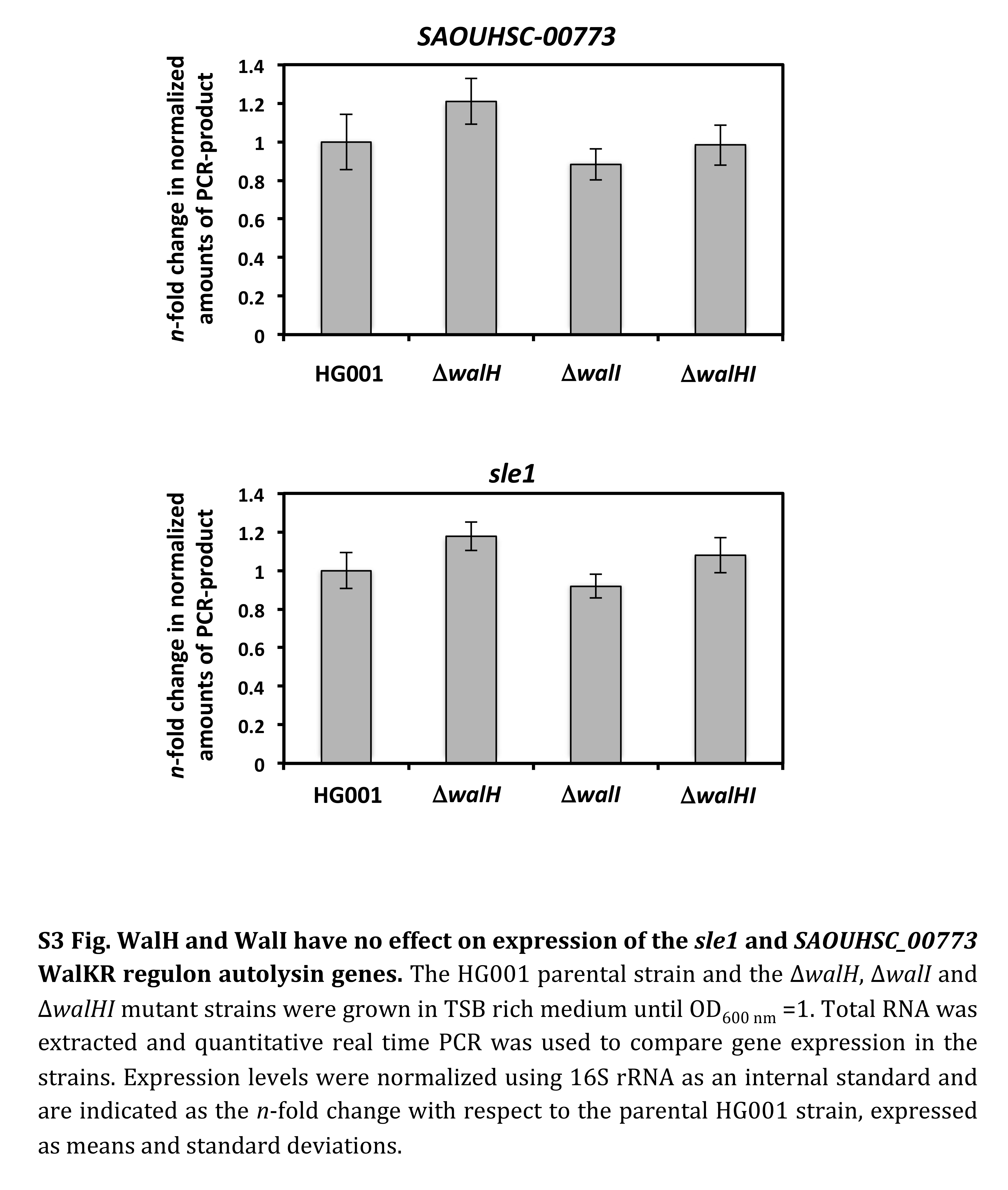

Supplement: S3 Fig — The HG001 parental strain and the ΔwalH, ΔwalI and ΔwalHI mutant strains were grown in TSB rich medium until OD600 nm = 1. Total RNA was extracted and quantitative real time PCR was used to compare gene expression in the strains. Expression levels were normalized using 16S rRNA as an internal standard and are indicated as the n-fold change with respect to the parental HG001 strain, expressed as means and standard deviations. (TIF) [file pone.0151449.s003.tif]

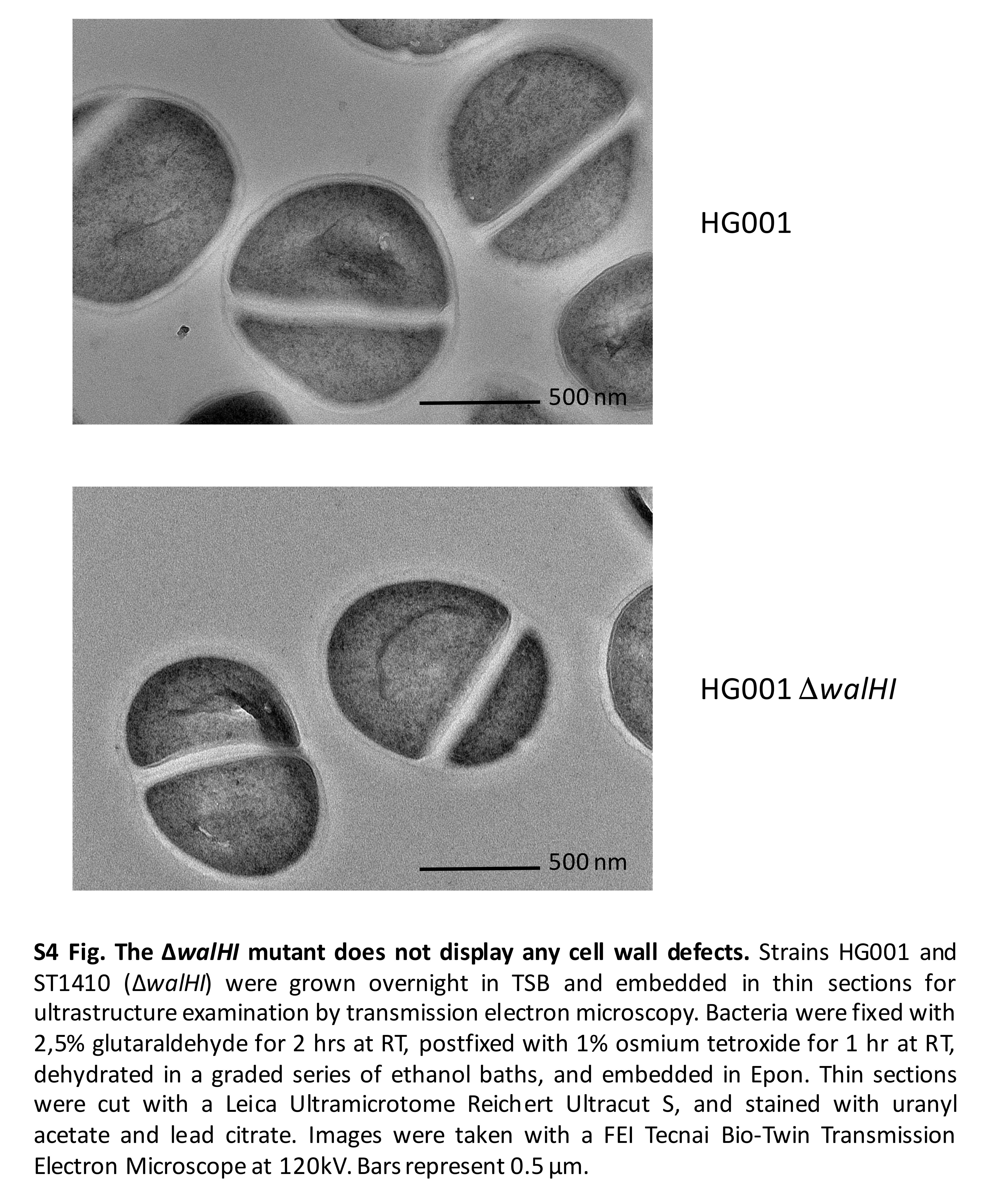

Supplement: S4 Fig — Strains HG001 and ST1410 (ΔwalHI) were grown overnight in TSB and embedded in thin sections for ultrastructure examination by transmission electron microscopy. Bacteria were fixed with 2,5% glutaraldehyde for 2 hrs at RT, postfixed with 1% osmium tetroxide for 1 hr at RT, dehydrated in a graded series of ethanol baths, and embedded in Epon. Thin sections were cut with a Leica Ultramicrotome Reichert Ultracut S, and stained with uranyl acetate and lead citrate. Images were taken with a FEI Tecnai Bio-Twin Transmission Electron Microscope at 120kV. Bars represent 0.5 μm. (TIF) [file pone.0151449.s004.tif]

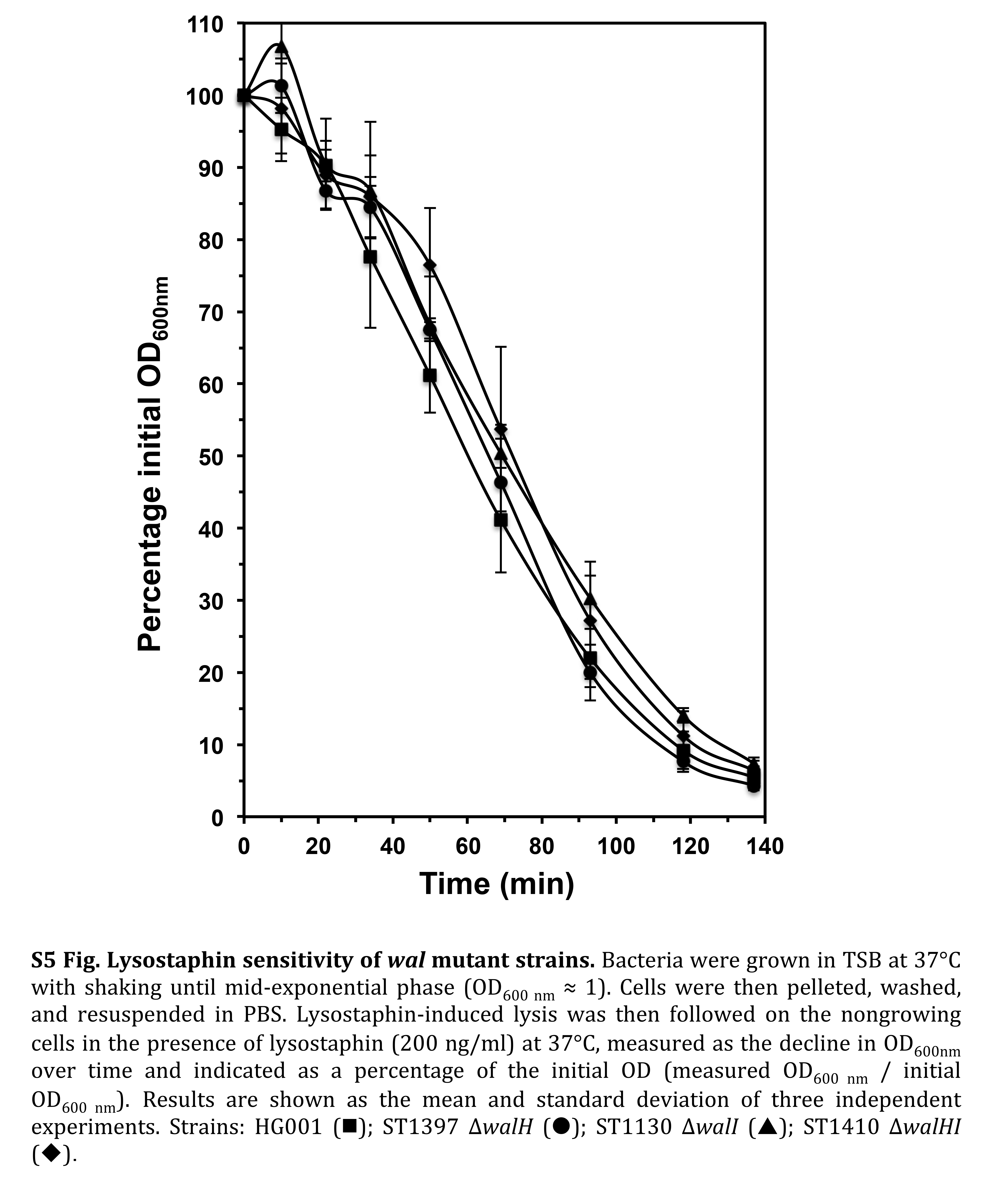

Supplement: S5 Fig — Bacteria were grown in TSB at 37°C with shaking until mid-exponential phase (OD600 nm ≈ 1). Cells were then pelleted, washed, and resuspended in PBS. Lysostaphin-induced lysis was then followed on the nongrowing cells in the presence of lysostaphin (200 ng/ml) at 37°C, measured as the decline in OD600nm over time and indicated as a percentage of the initial OD (measured OD600 nm / initial OD600 nm). Results are shown as the mean and standard deviation of three independent experiments. Strains: HG001 (▰); ST1397 ΔwalH (●); ST1130 ΔwalI (▲); ST1410 ΔwalHI (◆). (TIF) [file pone.0151449.s005.tif]

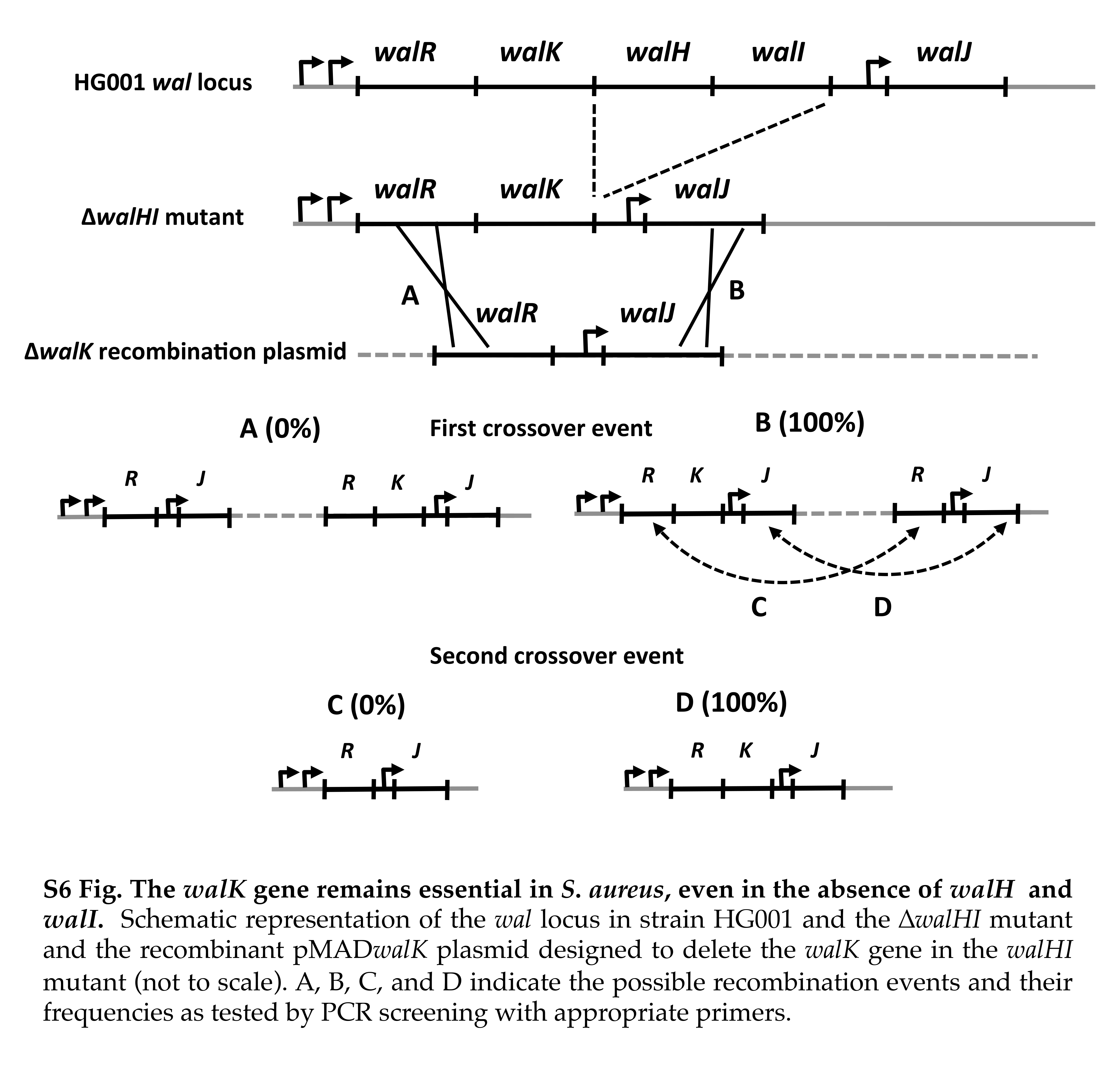

Supplement: S6 Fig — Schematic representation of the wal locus in strain HG001 and the ΔwalHI mutant and the recombinant pMADwalK plasmid designed to delete the walK gene in the walHI mutant (not to scale). A, B, C, and D indicate the possible recombination events and their frequencies as tested by PCR screening with appropriate primers. (TIF) [file pone.0151449.s006.tif]

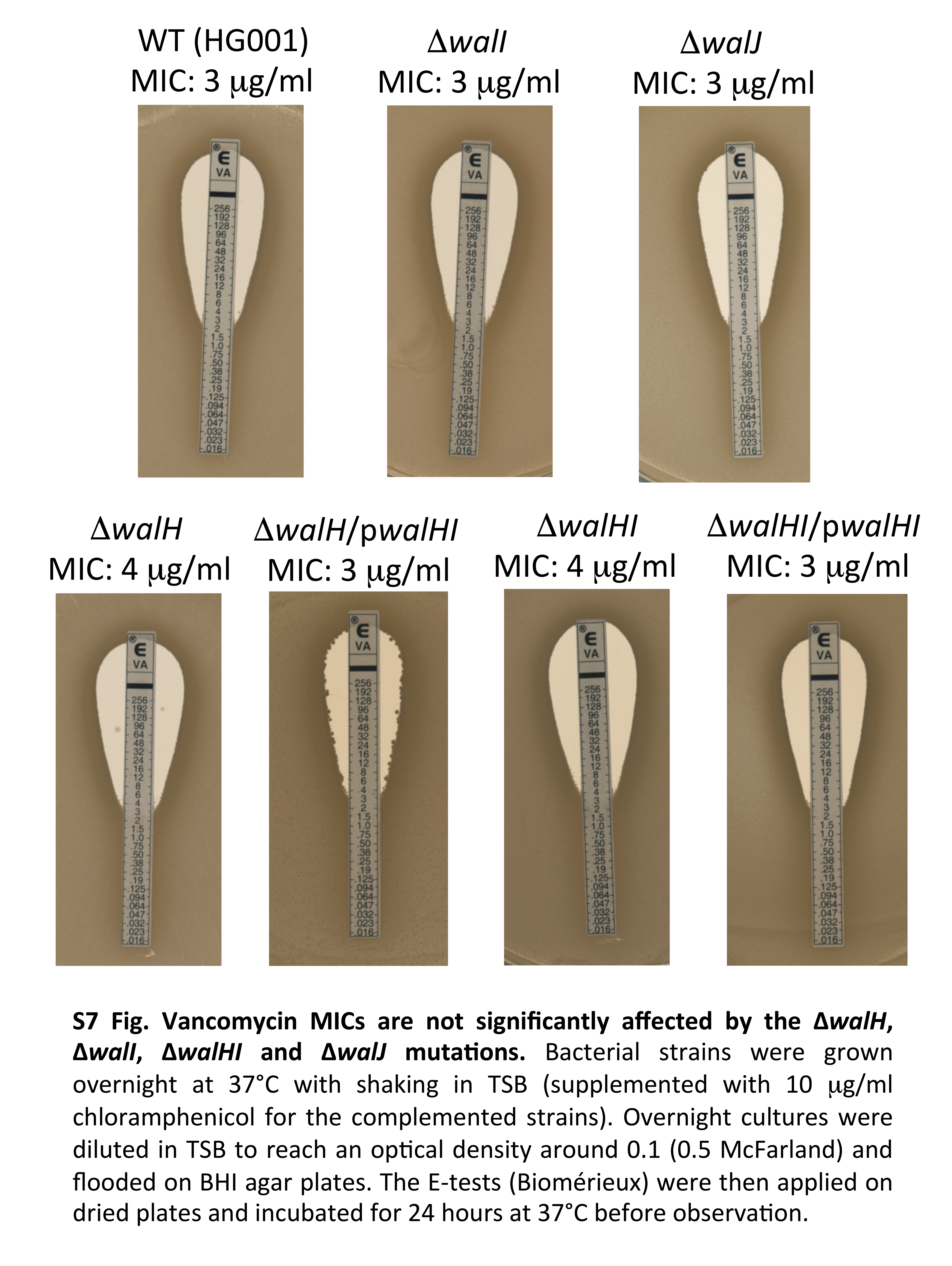

Supplement: S7 Fig — Bacterial strains were grown overnight at 37°C with shaking in TSB (supplemented with 10 μg/ml chloramphenicol for the complemented strains). Overnight cultures were diluted in TSB to reach an optical density around 0.1 (0.5 McFarland) and flooded on BHI agar plates. The E-tests (Biomérieux) were then applied on dried plates and incubated for 24 hours at 37°C before observation. (TIF) [file pone.0151449.s007.tif]

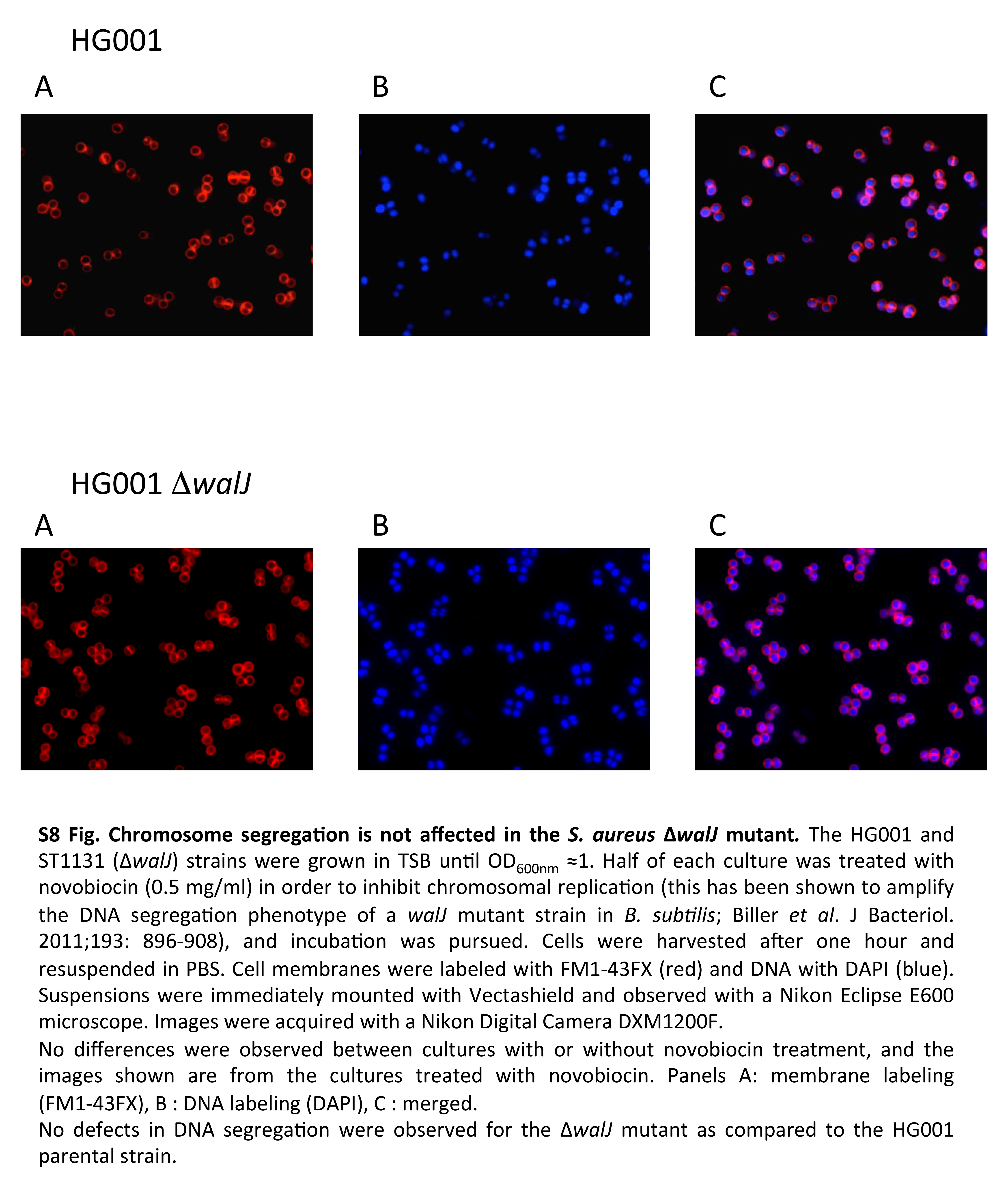

Supplement: S8 Fig — The HG001 and ST1131 (ΔwalJ) strains were grown in TSB until OD600nm ≈1. Half of each culture was treated with novobiocin (0.5 mg/ml) in order to inhibit chromosomal replication (this has been shown to amplify the DNA segregation phenotype of a ΔwalJ mutant strain in B. subtilis), and incubation was pursued. Cells were harvested after one hour and resuspended in PBS. Cell membranes were labeled with FM1-43FX (red) and DNA with DAPI (blue). Suspensions were immediately mounted with Vectashield and observed with a Nikon Eclipse E600 microscope. Images were acquired with a Nikon Digital Camera DXM1200F. No differences were observed between cultures with or without novobiocin treatment, and the images shown are from the cultures treated with novobiocin. Panels A: membrane labeling (FM1-43FX), B: DNA labeling (DAPI), C: merged. No defects in DNA segregation were observed for the ΔwalJ mutant as compared to the HG001 parental strain. (TIF) [file pone.0151449.s008.tif]

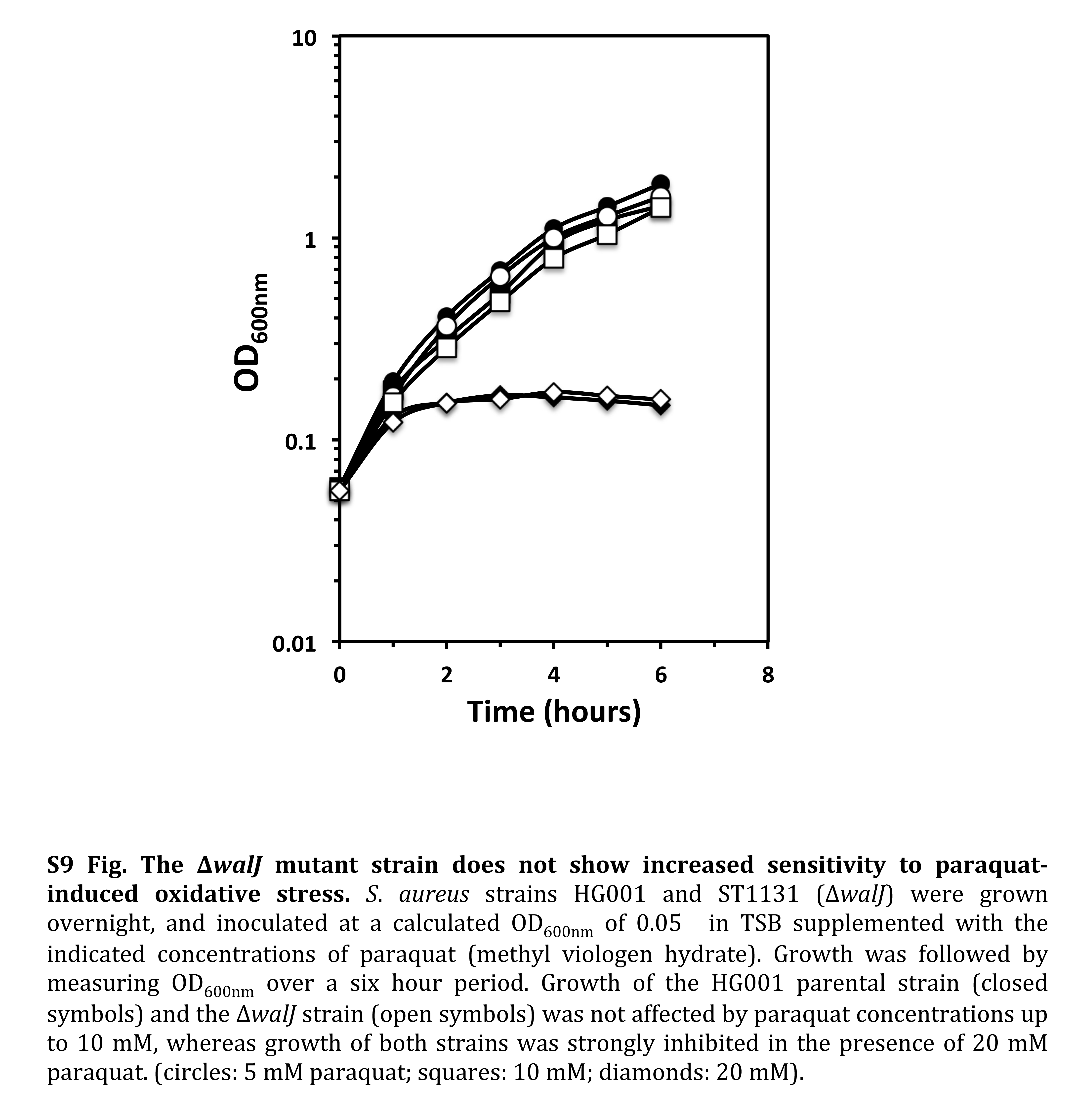

Supplement: S9 Fig — S. aureus strains HG001 and ST1131 (ΔwalJ) were grown overnight, and inoculated at a calculated OD600nm of 0.05 in TSB supplemented with the indicated concentrations of paraquat (methyl viologen hydrate). Growth was followed by measuring OD600nm over a six hour period. Growth of the HG001 parental strain (closed symbols) and the ΔwalJ strain (open symbols) was not affected by paraquat concentrations up to 10 mM, whereas growth of both strains was strongly inhibited in the presence of 20 mM paraquat. (circles: 5 mM paraquat; squares: 10 mM; diamonds: 20 mM). (TIF) [file pone.0151449.s009.tif]

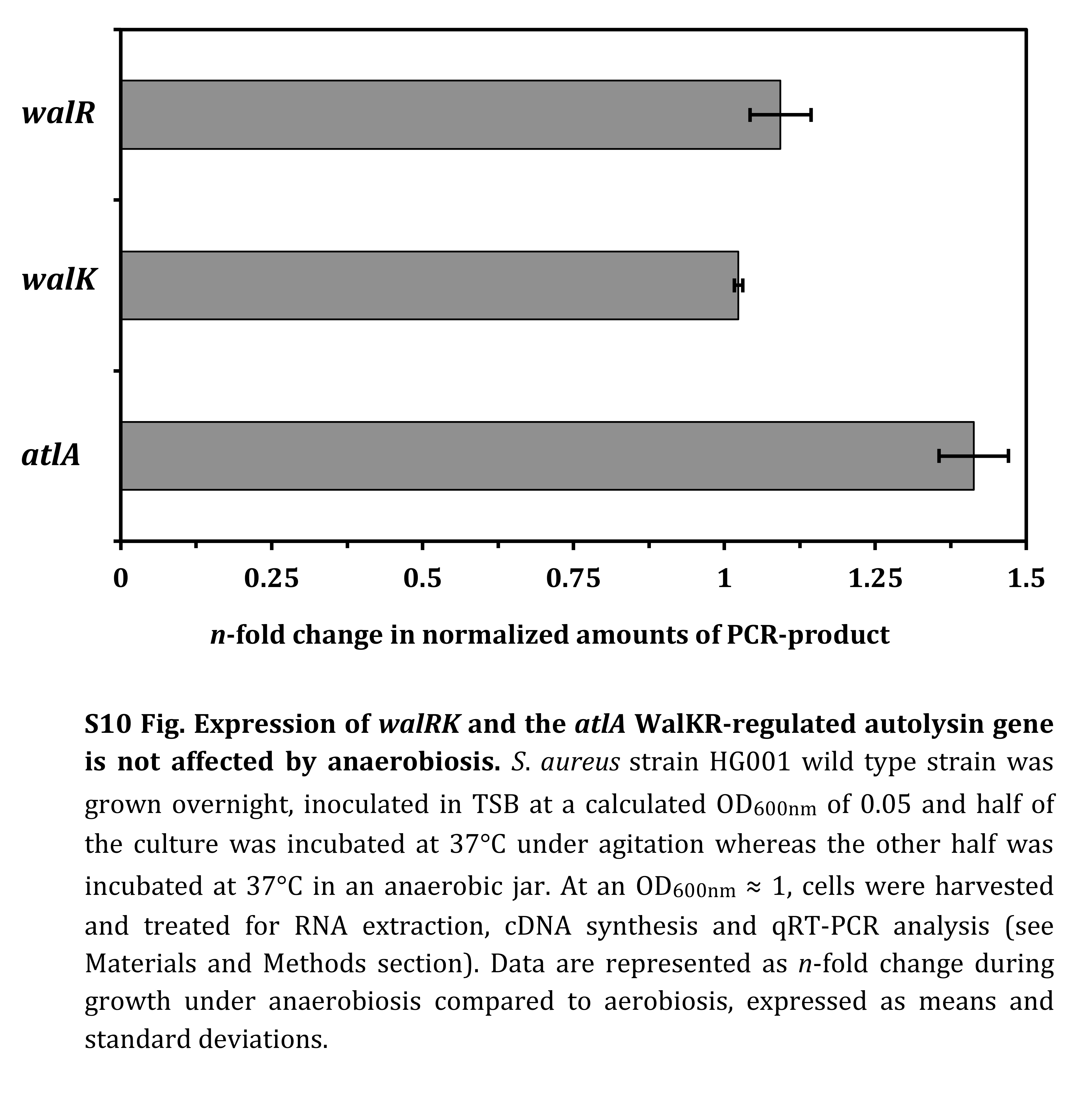

Supplement: S10 Fig — S. aureus strain HG001 wild type strain was grown overnight, inoculated in TSB at a calculated OD600nm of 0.05 and half of the culture was incubated at 37°C under agitation whereas the other half was incubated at 37°C in an anaerobic jar. At an OD600nm ≈ 1, cells were harvested and treated for RNA extraction, cDNA synthesis and qRT-PCR analysis (see Materials and Methods section). Data are represented as n-fold change during growth under anaerobiosis compared to aerobiosis, expressed as means and standard deviations. (TIF) [file pone.0151449.s010.tif]
