# Supplementary material for: Transcriptional Analysis and Subcellular Protein Localization Reveal Specific Features of the Essential WalKR System in Staphylococcus aureus
Source: PLoS One. 2016 Mar 21;11(3):e0151449. doi: 10.1371/journal.pone.0151449 (PMC4801191; doi:10.1371/journal.pone.0151449)
Supplement: S1 Table — (PDF) [file pone.0151449.s011.pdf]

Supporting Information Table S1

Sequence accession numbers for Wal proteins used in the phylogenomic analysis

TAXONOMIC REPRESENTATION					PROTEIN ACCESSION NUMBERS				
**TaxId**	**Class**	**Order**	**Family**	**Strain**	***walR***	***walK***	***walH***	***walI***	***walJ***
491915	Bacilli	Bacillales	Bacillaceae	*Anoxybacillus flavithermus* WK1	YP_002 317178	YP_002 317177	YP_002 317175	YP_002 317174	YP_002 317173
692420	Bacilli	Bacillales	Bacillaceae	*Bacillus amyloliquefaciens* DSM 7	YP_003 922455	YP_003 922454	YP_003 922453	YP_003 922452	YP_003 922451
720555	Bacilli	Bacillales	Bacillaceae	*Bacillus atrophaeus* 1942	YP_003 975507	YP_003 975506	YP_003 975505	YP_003 975504	YP_003 975503
649639	Bacilli	Bacillales	Bacillaceae	*Bacillus cellulosilyticus* DSM 2522	YP_004 097264	YP_004 097263	YP_004 097262	YP_004 097261	YP_004 097260
572264	Bacilli	Bacillales	Bacillaceae	*Bacillus cereus* 03BB102	YP_002 752825	YP_002 752824	YP_002 752823	YP_002 752822	YP_002 752821
66692	Bacilli	Bacillales	Bacillaceae	*Bacillus clausii* KSM-K16	YP_177 590	YP_177 589	YP_177 588	YP_177 587	YP_177 586
941639	Bacilli	Bacillales	Bacillaceae	*Bacillus coagulans* 2-6	YP_004 570389	YP_004 570388	YP_004 570387	YP_004 570386	YP_004 570385
315749	Bacilli	Bacillales	Bacillaceae	*Bacillus cytotoxicus* NVH 391-98	YP_001 377182	YP_001 377181	YP_001 377180	YP_001 377179	YP_001 377178
272558	Bacilli	Bacillales	Bacillaceae	*Bacillus halodurans* C-125	NP_24 4895	NP_24 4894	NP_24 4893	NP_24 4892	NP_24 4891
279010	Bacilli	Bacillales	Bacillaceae	*Bacillus licheniformis* DSM 13 = ATCC 14580	YP_081 404	YP_081 403	YP_081 402	YP_081 401	YP_081 400
592022	Bacilli	Bacillales	Bacillaceae	*Bacillus megaterium* DSM 319	YP_003 600394	YP_003 600393	YP_003 600392	YP_003 600391	YP_003 600390
398511	Bacilli	Bacillales	Bacillaceae	*Bacillus pseudofirmus* OF4	YP_003 426516	YP_003 426515	YP_003 426514	YP_003 426513	YP_003 426512
315750	Bacilli	Bacillales	Bacillaceae	*Bacillus pumilus* SAFR-032	YP_001 488893	YP_001 488892	YP_001 488891	YP_001 488890	YP_001 488889

439292	Bacilli	Bacillales	Bacillaceae	*Bacillus selenitireducens* MLS10	YP_003 701349	YP_003 701348	YP_003 701347	YP_003 701346	YP_003 701345
224308	Bacilli	Bacillales	Bacillaceae	*Bacillus subtilis*	NP_39 1921	NP_39 1920	NP_39 1919	NP_39 1918	NP_39 1917
412694	Bacilli	Bacillales	Bacillaceae	*Bacillus thuringiensis*	YP_897 645	YP_897 644	YP_897 643	YP_897 642	YP_897 641
315730	Bacilli	Bacillales	Bacillaceae	*Bacillus weihenstephanensis* KBAB4	YP_001 648030	YP_001 648029	YP_001 648028	YP_001 648027	YP_001 648026
235909	Bacilli	Bacillales	Bacillaceae	*Geobacillus kaustophilus* HTA426	YP_149 327	YP_149 326	YP_149 325	YP_149 324	YP_149 323
691437	Bacilli	Bacillales	Bacillaceae	*Geobacillus* sp. C56-T3	YP_003 672943	YP_003 672942	YP_003 672941	YP_003 672940	YP_003 672939
471223	Bacilli	Bacillales	Bacillaceae	*Geobacillus* sp. WCH70	YP_002 951312	YP_002 951311	YP_002 951310	YP_002 951309	YP_002 951308
550542	Bacilli	Bacillales	Bacillaceae	*Geobacillus* sp. Y412MC52	YP_004 134055	YP_004 134054	YP_004 134053	YP_004 134052	YP_004 134051
544556	Bacilli	Bacillales	Bacillaceae	*Geobacillus* sp. Y412MC61	YP_003 254565	YP_003 254564	YP_003 254563	YP_003 254562	YP_003 254561
420246	Bacilli	Bacillales	Bacillaceae	*Geobacillus thermodenitrificans* NG80-2	YP_001 127497	YP_001 127496	YP_001 127495	YP_001 127494	YP_001 127493
634956	Bacilli	Bacillales	Bacillaceae	*Geobacillus thermoglucosidasius* C56-YS93	YP_004 589814	YP_004 589813	YP_004 589812	YP_004 589811	YP_004 589810
444177	Bacilli	Bacillales	Bacillaceae	*Lysinibacillus sphaericus* C3-41	YP_001 700333	YP_001 700332	YP_001 700331	YP_001 700330	YP_001 700329
221109	Bacilli	Bacillales	Bacillaceae	*Oceanobacillus iheyensis* HTE831	NP_69 4374	NP_69 4373	NP_69 4372	NP_69 4371	NP_69 4370
272626	Bacilli	Bacillales	Listeriaceae	*Listeria innocua* Clip11262	NP_46 9660	NP_46 9661	NP_46 9662	NP_46 9663	NP_46 9664
637381	Bacilli	Bacillales	Listeriaceae	*Listeria monocytogenes* 08-5923	YP_003 415540	YP_003 415541	YP_003 415542	YP_003 415543	YP_003 415544

683837	Bacilli	Bacillales	Listeriaceae	*Listeria seeligeri* serovar 1_2b str. SLCC3954	YP_003 463509	YP_003 463510	YP_003 463511	YP_003 463512	YP_003 463513
386043	Bacilli	Bacillales	Listeriaceae	*Listeria welshimeri* serovar 6b str. SLCC5334	YP_848 462	YP_848 463	YP_848 464	YP_848 465	YP_848 466
262543	Bacilli	Bacillales	Bacillales Family XII. Incertae Sedis	*Exiguobacterium sibiricum* 255-15	YP_001 815493	YP_001 815492	YP_001 815491	YP_001 815490	YP_001 815489
360911	Bacilli	Bacillales	Bacillales Family XII. Incertae Sedis	*Exiguobacterium* sp. AT1b	YP_002 886123	YP_002 886124	YP_002 886125	YP_002 886126	YP_002 886127
358681	Bacilli	Bacillales	Paenibacillaceae	*Brevibacillus brevis* NBRC 100599	YP_002 775394	YP_002 775393	YP_002 775392	YP_002 775391	YP_002 775390
1036673	Bacilli	Bacillales	Paenibacillaceae	*Paenibacillus mucilaginosus* KNP414	YP_004 646297	YP_004 646296	YP_004 646295	YP_004 646294	YP_004 646293
349520	Bacilli	Bacillales	Paenibacillaceae	*Paenibacillus polymyxa* E681	YP_003 873213	YP_003 873212	YP_003 873211	YP_003 873210	YP_003 873209
324057	Bacilli	Bacillales	Paenibacillaceae	*Paenibacillus* sp. JDR-2	YP_003 014944	YP_003 014943	YP_003 014942	YP_003 014941	YP_003 014940
481743	Bacilli	Bacillales	Paenibacillaceae	*Paenibacillus* sp. Y412MC10	YP_003 246307	YP_003 246306	YP_003 246305	YP_003 246304	YP_003 246303
458233	Bacilli	Bacillales	Staphylococcaceae	*Macrococcus caseolyticus* JCSC5402	YP_002 559424	YP_002 559425	YP_002 559426	YP_002 559427	YP_002 559428
93062	Bacilli	Bacillales	Staphylococcaceae	*Staphylococcus aureus* subsp. *aureus* COL	YP_184 930	YP_184 931	YP_184 932	YP_184 933	YP_184 934
396513	Bacilli	Bacillales	Staphylococcaceae	*Staphylococcus carnosus* subsp. *carnosus* TM300	YP_002 635544	YP_002 635543	YP_002 635542	YP_002 635541	YP_002 635540
176280	Bacilli	Bacillales	Staphylococcaceae	*Staphylococcus epidermidis* ATCC 12228	NP_76 3573	NP_76 3574	NP_76 3575	NP_76 3576	NP_76 3577
279808	Bacilli	Bacillales	Staphylococcaceae	*Staphylococcus haemolyticus* JCSC1435	YP_251 932	YP_251 933	YP_251 934	YP_251 935	YP_251 937
698737	Bacilli	Bacillales	Staphylococcaceae	*Staphylococcus lugdunensis HKU09-01*	YP_003 472755	YP_003 472756	YP_003 472757	YP_003 472758	YP_003 472765
937773	Bacilli	Bacillales	Staphylococcaceae	*Staphylococcus pseudintermedius* HKU10-03	YP_004 150596	YP_004 150595	YP_004 150594	YP_004 150593	YP_004 150592

342451	Bacilli	Bacillales	Staphylococcaceae	*Staphylococcus saprophyticus* subsp. *saprophyticus* ATCC 15305	YP_300 111	YP_300 112	YP_300 113	YP_300 114	YP_300 115
866775	Bacilli	Lactobacillales	Aerococcaceae	*Aerococcus urinae* ACS-120-V-Col10a	YP_004 321849	YP_004 321848	YP_004 321847	YP_004 321846	YP_004 321844
208596	Bacilli	Lactobacillales	Carnobacteriaceae	*Carnobacterium* sp. 17-4	YP_004 373791	YP_004 373792	YP_004 373793	YP_004 373794	YP_004 373795
226185	Bacilli	Lactobacillales	Enterococcaceae	*Enterococcus faecalis* V583	NP_81 4922	NP_81 4923	NP_81 4924	NP_81 4925	NP_81 4926
940190	Bacilli	Lactobacillales	Enterococcaceae	*Melissococcus plutonius* ATCC 35311	YP_004 456565	YP_004 456564	YP_004 456563	YP_004 456562	YP_004 456561
891391	Bacilli	Lactobacillales	Lactobacillaceae	*Lactobacillus acidophilus* 30SC	YP_004 286406	YP_004 286407	YP_004 286408	YP_004 286409	YP_004 286410
695560	Bacilli	Lactobacillales	Lactobacillaceae	*Lactobacillus amylovorus* GRL 1112	YP_004 030896	YP_004 030897	YP_004 030898	YP_004 030899	YP_004 030900
387344	Bacilli	Lactobacillales	Lactobacillaceae	*Lactobacillus brevis* ATCC 367	YP_794 236	YP_794 237	YP_794 238	YP_794 239	YP_794 240
511437	Bacilli	Lactobacillales	Lactobacillaceae	*Lactobacillus buchneri* NRRL B-30929	YP_004 397399	YP_004 397400	YP_004 397401	YP_004 397402	YP_004 397403
321967	Bacilli	Lactobacillales	Lactobacillaceae	*Lactobacillus casei* ATCC 334	YP_807 964	YP_807 963	YP_807 962	YP_807 961	YP_807 960
748671	Bacilli	Lactobacillales	Lactobacillaceae	*Lactobacillus crispatus* ST1	YP_003 600545	YP_003 600546	YP_003 600547	YP_003 600548	YP_003 600549
353496	Bacilli	Lactobacillales	Lactobacillaceae	*Lactobacillus delbrueckii* subsp. *bulgaricus* 2038	YP_618 325	YP_618 326	YP_618 327	YP_618 328	YP_618 329
334390	Bacilli	Lactobacillales	Lactobacillaceae	*Lactobacillus fermentum* IFO 3956	YP_001 842831	YP_001 842832	YP_001 842833	YP_001 842834	YP_001 842835
324831	Bacilli	Lactobacillales	Lactobacillaceae	*Lactobacillus gasseri* ATCC 33323	YP_813 914	YP_813 915	YP_813 916	YP_813 917	YP_813 918
405566	Bacilli	Lactobacillales	Lactobacillaceae	*Lactobacillus helveticus* DPC 4571	YP_001 576655	YP_001 576656	YP_001 576657	YP_001 576658	YP_001 576659
633699	Bacilli	Lactobacillales	Lactobacillaceae	*Lactobacillus johnsonii*	YP_003	YP_003	YP_003	YP_003	YP_003

				FI9785	292277	292278	292279	292280	292281
1033837	Bacilli	Lactobacillales	Lactobacillaceae	*Lactobacillus kefiranofaciens* ZW3	YP_004 563440	YP_004 563441	YP_004 563442	YP_004 563443	YP_004 563444
644042	Bacilli	Lactobacillales	Lactobacillaceae	*Lactobacillus plantarum* JDM1	YP_003 061637	YP_003 061638	YP_003 061639	YP_003 061640	YP_003 061641
557436	Bacilli	Lactobacillales	Lactobacillaceae	*Lactobacillus reuteri* DSM 20016	YP_001 270632	YP_001 270633	YP_001 270634	YP_001 270635	YP_001 270636
568703	Bacilli	Lactobacillales	Lactobacillaceae	*Lactobacillus rhamnosus* GG	YP_003 172559	YP_003 172558	YP_003 172557	YP_003 172556	YP_003 172555
1069534	Bacilli	Lactobacillales	Lactobacillaceae	*Lactobacillus ruminis* ATCC 25644	YP_004 831310	YP_004 831311	YP_004 831312	YP_004 831313	YP_004 831314
314315	Bacilli	Lactobacillales	Lactobacillaceae	*Lactobacillus sakei* subsp. *sakei* 23K	YP_394 688	YP_394 689	YP_394 690	YP_394 691	YP_394 692
362948	Bacilli	Lactobacillales	Lactobacillaceae	*Lactobacillus salivarius* UCC118	YP_534 941	YP_534 942	YP_534 943	YP_534 944	YP_534 945
714313	Bacilli	Lactobacillales	Lactobacillaceae	*Lactobacillus sanfranciscensis* TMW 1.1304	YP_004 840437	YP_004 840438	YP_004 840439	YP_004 840440	YP_004 840441
278197	Bacilli	Lactobacillales	Lactobacillaceae	*Pediococcus pentosaceus* ATCC 25745	YP_805 260	YP_805 259	YP_805 258	YP_805 257	YP_805 256
349519	Bacilli	Lactobacillales	Leuconostocaceae	*Leuconostoc citreum* KM20	YP_001 727462	YP_001 727463	YP_001 727464	YP_001 727465	
762550	Bacilli	Lactobacillales	Leuconostocaceae	*Leuconostoc gasicomitatum* LMG 18811	YP_003 771775	YP_003 771776	YP_003 771777	YP_003 771778	
762051	Bacilli	Lactobacillales	Leuconostocaceae	*Leuconostoc kimchii* IMSNU 11154	YP_003 621415	YP_003 621416	YP_003 621417	YP_003 621418	
979982	Bacilli	Lactobacillales	Leuconostocaceae	*Leuconostoc* sp. C2	YP_004 706252	YP_004 706251	YP_004 706250	YP_004 706249	
1045854	Bacilli	Lactobacillales	Leuconostocaceae	*Weissella koreensis* KACC 15510	YP_004 726054	YP_004 726053	YP_004 726052	YP_004 726051	YP_004 726050
420889	Bacilli	Lactobacillales	Streptococcaceae	*Lactococcus garvieae* ATCC 49156	YP_004 778448	YP_004 778447			YP_004 778446
1359	Bacilli	Lactobacillales	Streptococcaceae	*Lactococcus lactis* subsp.	YP_001	YP_001			YP_001

				cremoris	031764	031763			031762
208435	Bacilli	Lactobacillales	Streptococcaceae	*Streptococcus agalactiae* 2603V_R	NP_68 7734	NP_68 7735			NP_68 7736
663952	Bacilli	Lactobacillales	Streptococcaceae	*Streptococcus dysgalactiae* subsp. *dysgalactiae* ATCC 27957	YP_002 996285	YP_002 996286			YP_002 996287
553482	Bacilli	Lactobacillales	Streptococcaceae	*Streptococcus equi* subsp. *equi* 4047	YP_002 746832	YP_002 746831			YP_002 746830
637909	Bacilli	Lactobacillales	Streptococcaceae	*Streptococcus gallolyticus* UCN34	YP_004 288496	YP_004 288495			YP_004 288494
467705	Bacilli	Lactobacillales	Streptococcaceae	*Streptococcus gordonii* str. Challis substr. CH1	YP_001 450078	YP_001 450079			YP_001 450080
365659	Bacilli	Lactobacillales	Streptococcaceae	*Streptococcus mitis* B6	YP_003 446288	YP_003 446287			YP_003 446286
511691	Bacilli	Lactobacillales	Streptococcaceae	*Streptococcus mutans* NN2025	YP_003 484505	YP_003 484506			YP_003 484507
927666	Bacilli	Lactobacillales	Streptococcaceae	*Streptococcus oralis* Uo5	YP_004 325948	YP_004 325949			YP_004 325950
760570	Bacilli	Lactobacillales	Streptococcaceae	*Streptococcus parasanguinis* ATCC 15912	YP_004 621863	YP_004 621862			YP_004 621861
936154	Bacilli	Lactobacillales	Streptococcaceae	*Streptococcus parauberis* KCTC 11537	YP_004 478477	YP_004 478479			YP_004 478480
981540	Bacilli	Lactobacillales	Streptococcaceae	*Streptococcus pasteurianus* ATCC 43144	YP_004 559570	YP_004 559569			YP_004 559568
189423	Bacilli	Lactobacillales	Streptococcaceae	*Streptococcus pneumoniae* 670-6B	YP_003 879216	YP_003 879217			YP_003 879218
1054460	Bacilli	Lactobacillales	Streptococcaceae	*Streptococcus pseudopneumoniae* ATCC BAA-960	YP_004 768485	YP_004 768484			YP_004 768483
160490	Bacilli	Lactobacillales	Streptococcaceae	*Streptococcus pyogenes* M1 GAS	NP_26 8802	NP_26 8803			NP_26 8804
1046629	Bacilli	Lactobacillales	Streptococcaceae	*Streptococcus salivarius*	YP_004	YP_004			YP_004

				57.I	727956	727955			727954
388919	Bacilli	Lactobacillales	Streptococcaceae	*Streptococcus sanguinis* SK36	YP_001 035505	YP_001 035504			YP_001 035503
391295	Bacilli	Lactobacillales	Streptococcaceae	*Streptococcus suis* 05ZYH33	YP_001 198725	YP_001 198724			YP_001 198723
299768	Bacilli	Lactobacillales	Streptococcaceae	*Streptococcus thermophilus* CNRZ1066	YP_141 523	YP_141 522			YP_141 521
218495	Bacilli	Lactobacillales	Streptococcaceae	*Streptococcus uberis* 0140J	YP_002 735123	YP_002 561898			YP_002 561899
